# Pastoralism in Northern Peru during Pre-Hispanic Times: Insights from the Mochica Period (100–800 AD) Based on Stable Isotopic Analysis of Domestic Camelids

**DOI:** 10.1371/journal.pone.0087559

**Published:** 2014-01-31

**Authors:** Elise Dufour, Nicolas Goepfert, Belkys Gutiérrez Léon, Claude Chauchat, Régulo Franco Jordán, Segundo Vásquez Sánchez

**Affiliations:** 1 Muséum national d’Histoire naturelle-CNRS, UMR 7209 Archéozoologie, Archéobotanique : sociétés, pratiques et environnements, Département Ecologie et Gestion de la Biodiversité, Paris, France; 2 CNRS-Paris 1, UMR 8096 Archéologie des Amériques, Nanterre, France; 3 Universidad Nacional de Trujillo, Trujillo, Peru; 4 Museo de Cao-Fundación Wiese, Lima, Peru; University of Florence, Italy

## Abstract

Llama (*Lama glama*) and alpaca (*Vicugna pacos*) are the only large domesticated animals indigenous to the Americas. Pastoralism occupies a fundamental economic, social and religious role in Andean life. Today, camelid livestock are confined to the ecozone of the *puna* (above 3,500 masl), while their presence on the Pacific coast during pre-Hispanic times is attested by archaeological skeletal remains. This study aims to document herding practices on the northern Peruvian coast during the Early Intermediate Period (200 BC-600 AD) by gaining insights into diet, location of breeding and mobility of archaeological camelids from the funerary and ritual contexts of two Mochica sites, Uhle Platform in Huacas de Moche and El Brujo. The three first early years and the long-term life histories of the animals were documented by the combined bulk analysis of bone collagen (δ^13^C_col_ and δ^15^N_col_) and bone structural carbonate (δ^13^C_bone_ and δ^18^O_bone_) and the serial analysis of structural carbonate of molar tooth enamel (δ^13^C_enamel_ and δ^18^O_enamel_). Mochica camelids were bred in the low and/or middle valleys, unlike their modern counterparts, who are restricted to highland *puna* C_3_ pastures. Archaeological camelids had diverse and complex life histories, usually with substantial maize foddering. An ontogenetic switch in diet and possible residential mobility during the course of life were identified for some specimens. Although the inference of geographic origin from δ^18^O_bone_ and δ^18^O_enamel_ values was limited because of the lack of understanding of the influence of environmental and biological factors, tooth enamel analysis has great potential for exploring camelid herding practices and Andean pastoralism. Our study suggested that Mochica herders adapted their practices to the difficult lowland environment and that herding practices were varied and not restricted to breeding at higher altitudes. The role of maize in different aspects of the economic life of the Mochicas is also underlined.

## Introduction

Andean pastoralism and the establishment of trade routes between different ecological zones, the so-called concept of Andean verticality, is one of the foundations for the emergence of complex societies in the pre-Hispanic world [1]. Alpacas (*Vicugna pacos*) and llamas (*Lama glama*) were domesticated 4,000–6,000 years ago, and experienced an intensification in livestock management from at least the end of the Early Horizon (900–400 BC) [2]. From then onwards, camelids have occupied a fundamental economic, social and religious role, both in pre-Hispanic and modern Andean cultures [3]. During the pre-Hispanic period, the llama was the only beast of burden and caravans providing goods to different ecological zones were crucial to the development of extensive trade networks [1], [4]–[6]. Textiles were manufactured from camelid wool and traded throughout the Andes, their meat was consumed, leather and bones served as raw materials to make tools and various ornaments, and dung was used as fuel. Domestic camelids were sacrificed and deposited into graves to fulfil various symbolic functions [7] and their entrails could be used to read omens. Finally, they were a symbol of prestige and a marker of identity [8].

Today, camelid husbandry is essentially restricted to the highlands [9], in an ecozone called the *puna* that comprises plateaus above 3,500 masl, and more precisely between 3,900 and 4,500 masl [10]. *Puna* conditons are characterized by hypoxia, low intra-annual variation in temperature but daily variation as high as 20°C and a higher precipitation rate than that of the lower western slopes of the Andes (between 200 and 1,500 mm per year [11]). Water is supplied by rain, snow and hail during the rainy season. The vegetation comprises wetlands such as *bofedales* (peatlands of the central Andes where Juncaceae species dominate and serve as the primary peat-formers) which are naturally conducive to llama and alpaca breeding. The optimum life conditions for llamas and alpacas vary according to the function the animal serves [12]. Alpaca herds devoted to wool production are raised in rich *bofedales* pasturelands at higher altitudes than llama herds. The presence of domestic camelids in remote and dramatically different habitats, like the Pacific coast – characterized by a higher mean temperature and higher seasonal variations in temperature than the *puna* and almost no precipitation – is attested by skeletal remains, textiles and iconography from at least 200 BC onwards [2], [8], [13]–[17]. It was formerly assumed that lowland camelids were not raised locally but instead were brought by caravans from the Andes shortly before being butchered or sacrificed [18]. This classical view is challenged by new zooarchaeological studies [7], [13], [19]. The northern coast (0–500 masl) of Peru has witnessed – among others – the development of rich and powerful cultures, such as the Mochica culture (100–800 AD), famous for the construction of monumental ceremonial centers and cultural artefacts such as vessels and metal ornaments [20]. Camelid skeletal remains were found in different Mochica contexts: domestic, burials, sacrifices, funerary banquets and offering deposits. Because of the arid to hyper arid conditions that prevail today, the Peruvian coast does not seem to provide favorable conditions to camelid herding, compared to the rich productive grasslands and wet *bofedales* of the *puna*. Only a few habitats located in the lower part of the valleys, fog oases (*lomas*) developing in the foothills, or irrigated fields could have provided pastures rich enough to sustain large herds. Maize foddering, diet supplements comprising marine resources (algae) or seasonal transhumance to mid-altitude valleys could have been used to compensate low food availability. Therefore, the breeding of flocks in an environment radically different from their original environment during pre-Hispanic times raises questions about the nature and the location of the dietary resources necessary to maintain animals and the seasonal or year-round nature of their keep. Furthermore, the presence of camelid remains on the north coast is not in itself indicative of local herding. With the exception of juveniles (under two years old) that have not been documented to travel long distances [21], the skeletal remains found in the northern coast could have belonged to animals first bred in the *puna* and subsequently brought to the coast. In order to answer these questions and document the management systems developed by coastal breeders it is essential to reconstruct camelid diet and zone of growth.

Measurements of stable isotopes of carbon (δ^13^C), nitrogen (δ^15^N), oxygen (δ^18^O) and strontium (^87^Sr/^86^Sr) in human and animal remains found in archaeological contexts are a primary source of information on the life history of humans and animals. The scope of isotopic analyses is still limited in South America compared to other regions but it is growing rapidly. The western slopes of the Andes present a wide variety of ecological zones [22] from the coast to the highlands, characterized by differences in physical parameters such as temperature and precipitation, as well as availability of different food resource categories. This results in relatively predictable geographical variations in dietary and environmental isotopic signatures across the landmass [11], [23], even though the baseline of environmental δ^18^O water data remains to be established on a local scale [11], [24], [25]. A number of paleodietary studies have been completed using δ^13^C and δ^15^N values that enable, for instance, the identification of maize consumption [26]–[30]. δ^13^C and δ^15^N values, as well as δ^18^O and ^87^Sr/^86^Sr values (that rely on climate and geology) can also be used to assess animal mobility between geographical areas that present distinct isotopic values [31]–[34].

Stable isotopic studies of past camelids in the Central Andes have mostly been conducted on bone collagen (reviewed by [34]) and more recently on bone structural carbonate [35], [36] of remains from various archaeological contexts, located at different elevations (lowlands, intermediate lands, highlands). They revealed some diversity in diet, pasturelands and foddering practices among sites and within sites. As bone tissue is constantly renewed during life, it provides a record of diet and habitat averaged out over the lifetime of the animal. Only tissues with accretional growth that do not undergo remodeling after deposition can record the chronology of the animal’s life history. Enamel of high-crowned teeth records events that occurred during the period of tooth formation and reconstructs this record through the isotopic profiles when an appropriate serial sampling protocol is applied.

The serial analysis of tooth enamel has been used successfully in different regions of the world to document herding practices [37], [38]. Preliminary data presented in Goepfert et al. [36] showed that this technique appears to be very promising in the Central Andean region. Based on these first data, this study aims to document herding practices on the northern Peruvian coast during the Early Intermediate Period (200 BC-600 AD). In order to provide insights into diet, location of breeding of domestic camelids and mobility patterns between the coast and higher elevations, the age of archaeological specimens from two Mochica sites (Uhle Platform in Huacas de Moche and El Brujo, La Libertad region) in the Moche and Chicama Valleys was determined and their stable isotopic composition was analysed and compared to that of modern camelids. The serial analysis of δ^13^C and δ^18^O values of structural carbonate from molar enamel (δ^13^C_enamel_ and δ^18^O_enamel_) was used to retrospectively record the animals’ early life histories. Enamel δ^13^C and δ^18^O values were compared to those of bone apatite (δ^13^C_bone_ and δ^18^O_bone_) and to δ^13^C and δ^15^N values for bone collagen (δ^13^C_col_ and δ^15^N_col_), to document diachronic changes in diet and growth location.

## Materials and Methods

### Geographical and Cultural Context

**Figure 1:**
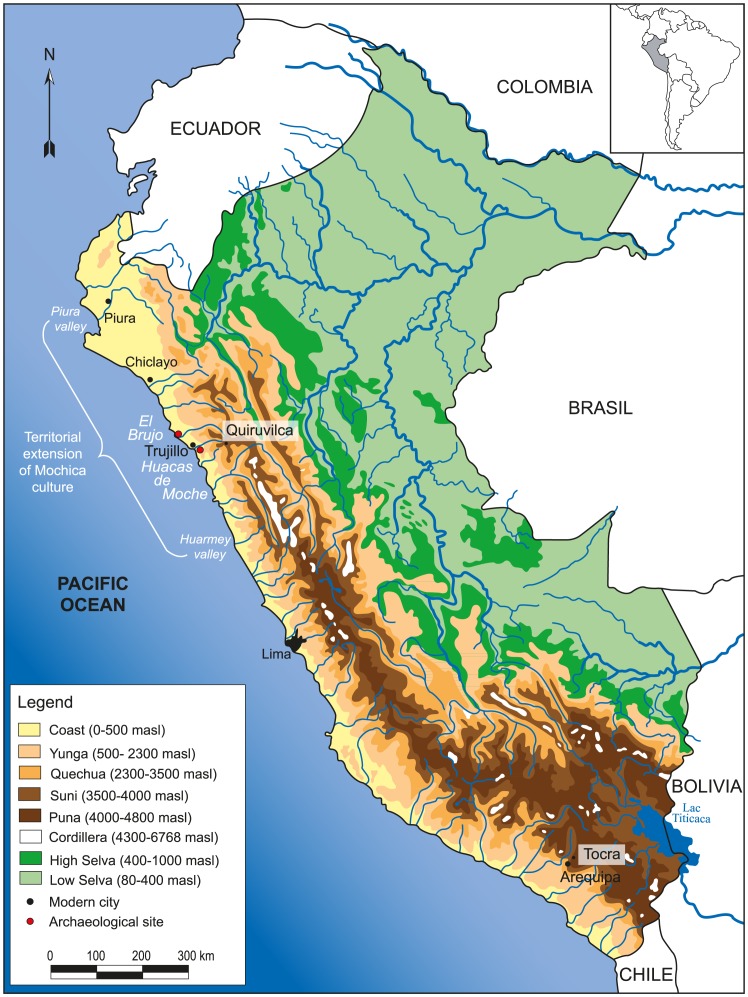
Topographic map of Peru with valleys and provenance of Mochica and modern domestic camelids.

**Figure 2:**
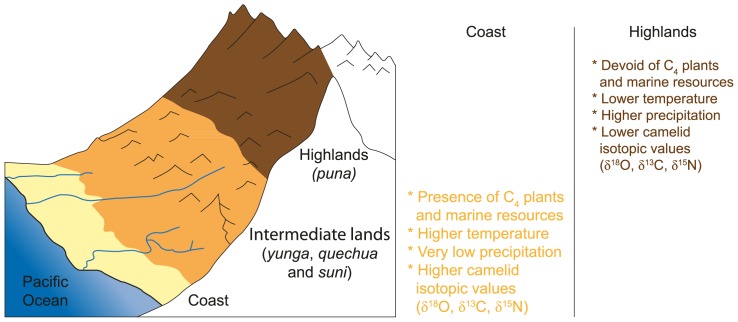
Central Andes ecozones and altitudinal environmental variations. Cross-section of the western slopes of the Central Andes with the main ecological zones considered in the study with expected trends in variations in environmental conditions and δ^13^C, δ^15^N and δ^18^O measured in domestic camelid tissues.

The Mochica territory ranged from the Piura Valley to the Huarmey Valley on the northern coast of Peru (Fig. 1). Mochica political power was not centralized, but was probably divided into independent centers located around the valleys [39] carved by rivers and streams between the coast and the Andean foothills along a northeast-southwest axis (Figs. 1–2). The Mochica people occupied the lower elevations of the Andes (0–500 masl), which presents contrasting environments. A relative chronology based on stylistic changes in the form and decoration of Mochica ceramic vessels (stirrup spout bottles) was defined by Larco Hoyle [40]. The sequence includes five successive phases: Moche I and II (100–200 AD), Moche III (200–450 AD), Moche IV (450–650 AD) and Moche V (650–800 AD).

At the latitude of the Moche region, the coast is a zone approximately 25 km wide, bordered to the west by a cool and rich ocean and to the east by the foothills of the Andes (Fig. 2). The coast is very arid and covered with sand and large dunes [41]. With less than 20 mm of rain per year, surface waters result mostly from the winter mists called *garúa,* which occur between May and December, and the resurgence of the underground water network [42]. *Lomas* develop on the foothills between 200 and 1,000 masl and present specific vegetation which is particularly attractive to wildlife and game. The northern boundary of the *lomas* is located on the foothills of the Cerro Campana, a few kilometers from the modern city of Trujillo. The rivers and the valleys are the most active and dynamic components of the coastal environment and have been classified as oases by Dollfus [43]. With the exception of *lomas*, river mouths and valleys, the arid desert environment of the coast is not conducive to agriculture and permanent domestic animal herding. The sustainability of diversified agriculture was made possible by the development of a complex irrigation system formed of canals, which undoubtedly constituted one of the bases for the expansion of the Mochicas into each valley [44], [45].

**Figure 3:**
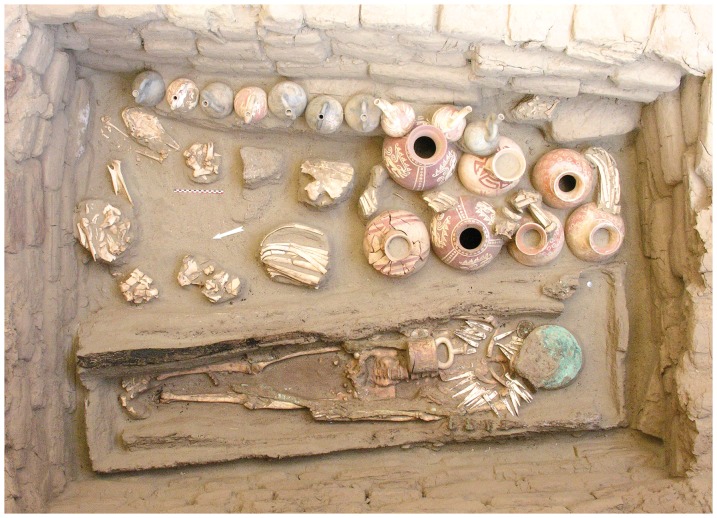
Tomb 48(Huacas de Moche) with artefacts and domestic camelid offerings.

The site of Huacas de Moche is located on the left bank of the Moche Valley, 4 km from Trujillo (Fig. 1). It comprises two pyramids, the Huaca del Sol and Huaca de la Luna, which are now separated by a vast desert plain which covers an ancient city, the Urban Area, which included domestic sectors and craft workshops. The Uhle Platform, a 75 m long and 25 m wide structure, is located at the bottom of the west side of the Huaca de la Luna. Several excavations were conducted at the Uhle Platform by the Moche International Programme between 1999 and 2008 [46]–[51]. They led to the discovery of 57 Mochica graves, several human sacrifice areas and large ceremonial deposits. Camelids were found in 42 graves (Fig. 3) [13].

The site of El Brujo is located 60 km north of Trujillo, close to the village of Magdalena de Cao, in the lower part of the Chicama Valley (Fig. 1), along the ocean shore [52], [53]. It comprises two Mochica pyramids, The Huaca Cao Viejo and the Huaca El Brujo. Like Huacas de Moche, El Brujo was one of the major religious centers on the northern coast of Peru. Excavations have been conducted at El Brujo since 1990 by the El Brujo Archaeological Project [52]–[54]. Tens of Mochica burials have been discovered [52]–[57], of which eleven contained camelid remains [13].

### Material

#### Ethic statement

All necessary permits were obtained for the described study, which complied with all relevant regulations. Permissions were obtained from the Ministerio de Cultura of Peru to study archaeological remains.

#### Archaeological material

Species-specific determination of camelids based on the study of skeletal remains is theoretically possible but proved to be difficult. The phalange is usually the preferred anatomical element [58], but is of limited use here as the presence of this bone in the archaeological site is restricted to adults and it cannot be used to separate hybrid specimens. We therefore used tooth shape [59] and the size of skeletal remains to discriminate between large (*Lama* sp.) camelids and small camelids (*Vicugna* sp.). Only *Lama* sp. were identified in our two archaeological sites and were probably llamas. However, this does not preclude the presence of alpacas on the coast during pre-Hispanic times. Eight archeological camelid specimens curated at the site Museum of the Huacas de Moche (Moche, La Libertad, Peru) were selected from five graves and from one ceremonial context of the Uhle Platform (Table 1) [46]–[49], [51]. These contexts were dated from the Moche I to Moche IV phases. One piece of bone was selected from the mandible, the skull or a phalanx for six out of the eight individuals for bone collagen extraction (Table 2). All the permanent molars present in each hemi-mandible were extracted. Depending on the stage of tooth eruption, one (UH-4, UH-7), two (UH-3, UH-5, UH-9) or the three molars (UH-8, UH-10, UH-11) were present (Table3). δ^18^O_enamel_ values for the M2 and M3 of three individuals were previously published in Goepfert et al. [36]. Four specimens (*Lama* sp.), curated at the site Museum of El Brujo (Magdalena de Cao, La Libertad, Peru), were selected from four graves at El Brujo (Table 1). Three graves were located in the Huaca Cao Viejo, the fourth was situated near the ceremonial place (Ceremonial Wells 2). These contexts were dated from the Moche III and IV phases. A piece of bone from the mandible or the pelvis was collected for each specimen for bone collagen extraction (Table 2). One (EBT2-1995, EBT6-1994) or three (EBT2-1998, EBE1-1995) permanent molars were present on each jaw (Table 3).

**Table 1:** Origin, context and individual age of modern and archaeological and modern domestic camelids.

Specimen	Region	Origin/Site	Context		Species	Age
Areq-1	Arequipa	Tocra	Modern		*Vicugna pacos*	9–11 years
Areq-2	Arequipa	Tocra	Modern		*Vicugna pacos*	0–3 months
Areq-3	Arequipa	Tocra	Modern		*Lama glama*	3–6 months
Quiru-1	La Libertad	Quirivulca	Modern		*Vicugna pacos*	6 year 3 months-6 years 9 months
UH-3	La Libertad	Uhle Platform	Tomb 14	Moche IV	*Lama sp.*	2 years 3 months
UH-4	La Libertad	Uhle Platform	Tomb 22	Moche IV	*Lama sp.*	1 year 9 months-2 years
UH-5	La Libertad	Uhle Platform	Tomb 48	Moche III	*Lama sp.*	3 years 3 months
UH-7	La Libertad	Uhle Platform	Tomb 55	Moche I	*Lama sp.*	1 years 3 months-2 years
UH-8	La Libertad	Uhle Platform	Element 21	Moche I	*Lama sp.*	6 years
UH-9	La Libertad	Uhle Platform	Element 21	Moche I	*Lama sp.*	3 years-3 years 6 months
UH-10	La Libertad	Uhle Platform	Element 21	Moche I	*Lama sp.*	4 years 6 months- 4 years 9 months
UH-11	La Libertad	Uhle Platform	Tumb 16	Moche III	*Lama sp.*	11–13 years
EBT2-1995	La Libertad	El Brujo	Tomb 2/Well 2	n/d	*Lama sp.*	1 year 3 months-2 years
EBT6-1994	La Libertad	El Brujo	Tomb 6/Huaca Cao Viejo	Moche III–IV	*Lama sp.*	<3 years
EBT2-1998	La Libertad	El Brujo	Tomb 2/Huaca Cao Viejo	n/d	*Lama sp.*	7 years-7 years 6 months
EBE1-1995	La Libertad	El Brujo	Tomb 1/Huaca Cao Viejo	n/d	*Lama sp.*	9 years 6 months

n/d : not determinate.

**Table 2:** δ^13^C (‰ VPDB) and δ^15^N (‰ AIR) values of bone collagen and δ^13^C and δ^18^O values (‰ VPDB) of bone structural carbonate of modern and archaeological domestic camelids.

Specimen	Bone collagen							Carbonate			
	δ^13^C (‰VPDB)	δ^15^N (‰ AIR)	%C	%N	C:N	wt %	%C4	δ^13^C (‰VPDB)	δ^18^O (‰VPDB)	% C_4_	Δ δ^13^C (‰VPDB)
Areq-1	−20.8	3.1	43.3	15.7	3.2	21.7	−	−12.3	−7.7	−	8.5
Areq-2	−20.2	5.1	43.7	16.3	3.1	17.3	−	−14.2	−10.0	−	6.0
Areq-3	−20.3	4.6	44.1	16.4	3.1	19.8	−	−14.7	−8.3	−	5.6
Quiru-1	−20.6	3.8	41.6	14.9	3.3	21.4	−	−13.6	−4.2	−	7.0
UH-3	−	−	−	−	−	−	−	−	−	−	−
UH-4	−	−	−	−	−	−	−	−	−	−	−
UH-5	−	−	−	−	−	−	−	−7.7	−0.5	45	−
UH-7	−	−	−	−	−	−	−	−9.3	1.3	34	−
UH-8	−	−	−	−	−	−	−	−7.8	0.7	44	−
UH-9	−	−	−	−	−	−	−	−7.8	0.7	44	−
UH-10	−	−	−	−	−	−	−	−8.9	−0.7	36	−
UH-11	−	−	−	−	−	−	−	−7.9	−3.4	44	−
EBT2-1995		−	−	−	−	−	−	−5.8	−2.1	59	−
EBT6-1994	−14.8	8.5	43.2	16.1	3.1	6.5	43	−7.2	1.5	49	−7.2
EBT2-1998	−13.6	10.0	44.0	16.3	3.1	17.0	51	−5.7	−1.9	59	−7.9
EBE1-1995	−13.2	7.4	41.2	15.4	3.1	1.9	54	−5.6	−1.9	60	−7.6

δ^13^C values of modern specimens were corrected by +1.5 ‰ for the Suess effect [108]. Proportion of C_4_ (%) in the diet was reconstructed using mean estimated δ^13^C values of pure-C_3_ and pure-C_4_ feeders.

**Table 3:** Summary of δ^13^C and δ^18^O values (‰ VPDB) of enamel structural carbonate of molars of archaeological and modern camelids.

Specimen	Teeth		n	δ^13^C (‰VPDB)					% C_4_		δ^18^O (‰VPDB)				
				mean	SD	min.	Max.	range			mean	SD	min.	max.	range
Areq-1	M1		5	−10.4	0.9	−11.2	−8.9	2.3		−	−7.6	1.3	−9.7	−6.5	3.2
	M2		9	−10.5	0.4	−11,0	−10.1	0.9		−	−8.8	0.2	−9.1	−8.5	0.6
	M3		15	−9.8	0.6	−10.6	−8.9	1.7		−	−8.2	1.4	−10.1	−5.4	4.7
Areq-3	Pd4		7	−12.1	0.8	−13.6	−11.5	2.1		−	−7.1	1,0	−8.5	−5.7	2.8
Quiri−1	M1		4	−10.7	0.4	−11.3	−10.4	0.9		−	−2.0	0.6	−2.7	−1.3	1.4
	M2		7	−9.6	0.2	−10,0	−9.4	0.6		−	−4.2	0.9	−5.5	−2.9	2.6
	M3		12	−10.8	0.3	−11.4	−10.5	0.9		−	−3.5	1.1	−4.6	−1.5	3.1
UH-3	M1		14	−3.7	0.2	−4.1	−3.4	0.7		57	−3.1	0.2	−3.6	−2.9	0.7
	M2		27	−2.4	0.5	−3.4	−1.5	1.9		66	−1.9	0.5	−2.8	−1.2	1.6
UH-4	M1		14	−9.7	0.2	−10.0	−9.2	0.8		16	−0.4	0.8	−1.4	1.0	2.4
UH-5	M1		20	−7.8	0.7	−8.7	−6.6	2.1		29	−1.0	0.2	−1.4	−0.6	0.8
	M2		34	−5.9	0.6	−6.7	−4.9	1.8		42	0.4	0.3	−0.4	0.9	1.3
UH-7	M1		16	−10.0	0.4	−10.5	−9.2	1.3		14	−0.3	1.0	−2.2	1.0	3.2
UH-8	M1		5	−9.3	0.1	−9.5	−9.2	0.3		19	1.6	0.2	1.3	1.8	0.5
	M2		25	−7.0	0.5	−7.8	−6.2	1.6		34	0.1	0.3	−0.8	0.4	1.2
	M3		17	−6.1	0.3	−6.5	−5.3	1.2		41	−0.7	0.3	−1.6	−0.4	1.2
UH-9	M1		12	−7.2	0.5	−8.3	−6.7	1.6		33	0.8	0.7	−0.4	1.6	2.0
	M2		25	−6.2	0.2	−6.6	−5.9	0.7		40	0.1	0.3	−0.4	0.9	1.3
UH-10	M1		10	−7.6	0.5	−8.2	−7.0	1.2		30	−0.6	0.4	−0.9	0.4	1.3
	M2		22	−6.6	0.3	−7.3	−6.1	1.2		37	−1.3	0.5	−2.0	−0.3	1.7
	M3		19	−6.0	0.7	−7.1	−4.9	2.2		41	−1.7	0.5	−2.3	−0.9	1.4
UH-11	M1		6	−6.1	0.2	−6.4	−5.9	0.5		41	1.3	0.2	1.0	1.5	0.5
	M2		22	−3.6	0.5	−4.4	−3.1	1.3		58	0.6	0.3	0.1	1.2	1.1
	M3		35	−4.6	0.8	−5.9	−3.1	2.8		51	−2.2	0.8	−3.4	−0.8	2.6
EBT2-1995	M1		15	−2.1	2.0	−5.5	−0.1	5.4		68	−0.5	0.9	−2.2	0.6	2.8
EBT6-1994	M1		16	−7.3	1.9	−9.9	−5.0	4.9		32	1.7	1.0	0.6	3.1	2.5
EBT2-1998	M1		7	−6.3	0.2	−6.6	−6.0	0.6		39	0.1	0.1	0.0	0.3	0.3
	M2		15	−4.6	0.9	−6.7	−3.4	3.3		51	−1.9	0.6	−2.9	−1.2	1.7
	M3		21	−3.6	0.8	−4.6	−2.0	2.6		58	−1.3	0.4	−2.0	−0.8	1.2
EBE1-1995	M1		8	−7.1	0.2	−7.6	−6.8	0.8		34	−0.2	0.2	−0.4	0.1	0.5
	M2		13	−8.1	0.2	−8.3	−7.6	0.7		27	−1.0	0.1	−1.2	−0.9	0.3
	M3		18	−1.4	0.4	−1.9	−0.7	1.2		73	−2.3	0.2	−2.6	−2.1	0.5

δ^13^C values of modern specimens were corrected by +1.5 ‰ for the Suess effect [108]. Proportion of C_4_ (%) in the diet is reconstructed using mean estimated δ^13^C values of pure-C_3_ and pure-C_4_ feeders.

#### Modern material

Teeth and bone from modern butchered animals (Table 1; Fig. 1) were kindly obtained with the permission of herders, Cayetano Mamani (Tocra, Arequipa, Peru) and Santos Mantilla (Quiruvilca, La libertad, Peru). Two alpacas and one llama from the southern region (National reserve of Salinas y Aguada Blanca, Tocra, Arequipa region at 4,200 masl) and one modern alpaca from the northern region (Quiruvilca, La Libertad region at 4,100 masl) were selected. While Quiruvilca is located in the upper Moche Valley, an area which could have provided camelids for the Mochicas, Tocra is situated outside the potential area of interaction between the Mochicas and highland camelid breeders. However, the two *puna* regions present similar environmental characteristics and vegetation formed of *bofedales*. In both locations, modern livestock are raised for meat production and wool and spend their whole life feeding on natural pastures. They thus appear well suited to exploring variations in isotopic values of camelids originating from the *puna* ecological zone. δ^18^O_enamel_ values for the M2 and M3 of Areq-1 have been described in Goepfert et al. [36]. Mandibles were defleshed and cleaned by boiling. A piece of mandibular bone was collected from the four individuals for bone collagen and bone apatite analyses (Table 2). Depending on the specimen, the three permanent molars (Areq-1, Quiru-1), or the Pd4 premolar (Areq-3) were extracted from the hemi-mandible (Table 3).

#### Individual age estimate and life history record

The age of studied specimens is rarely – if ever – mentioned in the literature, even though it appears essential for the interpretation of each specimen’s life history from the isotopic analysis of its tissues. Age-at-death of modern and archaeological animals was estimated by the observation of tooth wear and eruption stages established by Wheeler [59]. Age varied between 1 year and 9 months and 13 years and between 3 months and 6 years 9 months, respectively for archaeological and modern camelids (Table 1).

Bone and enamel form at different stages of an individual’s life and present different metabolic activities. Bone is constantly remodeled. Cogenetic bone collagen and structural carbonates remodel together and both provide a long-term isotopic signal. In humans, bone turnover varies between 10 to 30 years, depending on the age of the individual [60], [61]. Our sample comprises both young and old specimens. Therefore, the bulk analysis of bone collagen and bone apatite represents on average a few years (for young individuals) and the entire lifespan (for old individuals). Tooth enamel is metabolically inert. It is not remodeled once formed and integrates an individual’s life history over a fixed period of time. Camelids have hypsodont teeth characterized by a high crown with extended incremental growth [62]. The mineralization process is complex and composed of two different stages: growth and maturation. The duration of mineralization has been determined for domestic sheep (*Ovis aries*) and cattle (*Bos Taurus*) [63]–[65]. The schedule of crown growth completion, which is fixed for a given species, was estimated from the time of tooth eruption for domestic camelids. Camelid tooth eruption age is 6 to 9 months for the M1, 17 to 24 months for the M2 and 33 to 44 months for the M3 [59]. The beginning of crown formation is more difficult to assess. In comparison, in domestic sheep, the M1 crown starts to form in utero, the M2 during the second month after birth and the M3 when the lamb is 11 months old [65], [66]. If camelid molar growth follows the same pattern, the analysis of molars M1 to M3 should theoretically allow us to reconstruct a continuous record of the first 3–3.5 years of life of these animals. However, the upper part of each tooth is often worn and therefore the first part of the record of each tooth is missing. In addition, the consumption of milk can influence the isotopic values of tissue in formation [67]. As the first permanent molar grows during the early childhood, breastfeeding can potentially confound its isotopic signal.

### Sampling and Analysis of Camelid Tissues

Enamel and bone surfaces were mechanically cleaned to remove soft tissue, adherent soil or other contaminants by abrasion with a tungsten drill bit. Mechanical cleaning also detached the adherent cement on teeth and the outmost layer of bone, which is most susceptible to diagenetic contaminants. Cleaned bone samples were hand ground using an agate mortar and pestle and passed through a series of sieves. The finer fraction (<300 µm) was selected for determining δ^18^O_bone_ and δ^13^C_bone_ values.

**Figure 4:**
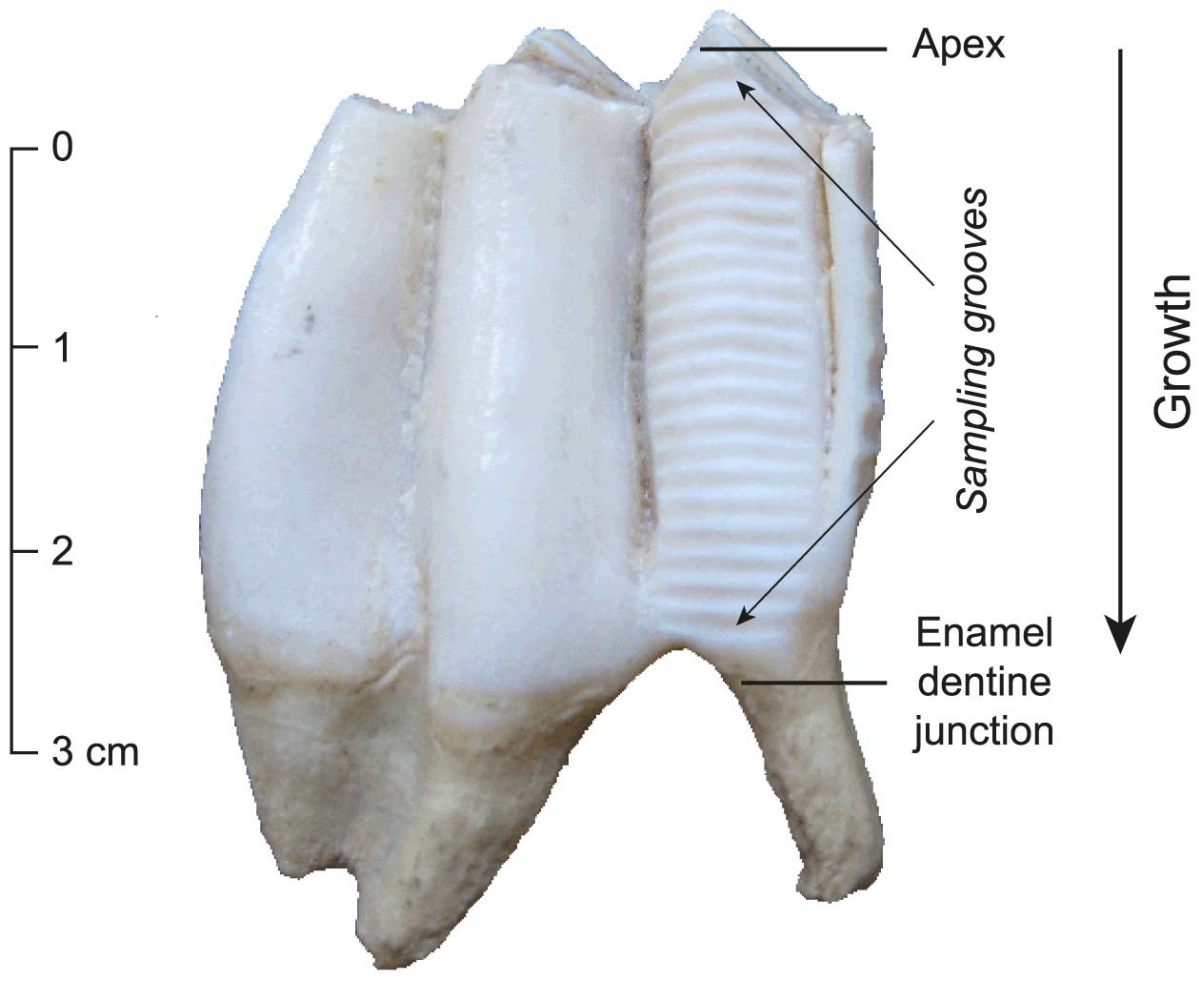
Sampling of molar enamel. Permanent M3 molar from specimen EBT2–1998 from El Brujo showing the series of enamel samples (grooves) that have been drilled along the highest lobe of the crown, from the apex to the enamel-root junction.

Crown height was measured for each tooth and the highest lobe was selected for sampling. Sequential sampling perpendicularly to the crown growth axis, from the apex to the enamel-dentine junction, was performed using a diamond drill bit. For El Brujo and modern camelid molars, each sample was a 1-mm-wide groove, taken from the buccal side through the whole thickness of the enamel layer (Fig. 4). Uhle Platform camelid molars were fragile and prone to breakage during this direct sampling. The enamel layer on the buccal face was thus first separated from the rest of the crown. Any adhering dentine was removed using a tungsten carbide drill bit and then the sequential sampling of the enamel was conducted as described previously to obtain an intra-tooth enamel sample series. Between 5 and 35 samples (5–11 mg) were obtained per tooth, depending on crown height.

Bone organic matter was eliminated using a NaOCl (2–3%) treatment. Then, both enamel and bone powder samples were purified with 0.1 M acetic acid (4 h, 0.1 ml mg^−1^) and 1M acetic acid (1 h, 0.1 ml mg^−1^), respectively, due to the higher susceptibility of bone to diagenetic contamination. The acetic acid treatment was used to remove exogenous carbonate and was carried out on both archaeological and modern specimens to ensure consistency when comparing results. All samples were then rinsed at least five times and oven-dried at 50°C. Bioapatite samples weighing 600 µg were reacted with 100% phosphoric acid at 70°C for 4 min in a Kiel IV device, interfaced with a Delta V Advantage isotope ratio mass spectrometer. Analytical precision was ±0.03 ‰ for δ^13^C (1σ) and ±0.05 ‰ for δ^18^O (1σ), based on the repeated analysis of an internal calcite standard (Marble LM), previously calibrated against NBS-19.

Collagen was extracted from the coarser grain size fraction (0.3–0.7 mm), following Longin’s [68] protocol, modified by Bocherens et al. [69]. About 200 mg of powdered bone was demineralized in a 1 M HCl solution for 20 min at room temperature then filtered through a MF-Millipore 5 µm filter. Potential contamination of fulvic and humic acids was removed by a 0.125 M NaOH (20 hours) treatment. After filtration, collagen was solubilized into a 0.02 M HCl (pH = 2) solution at 100°C for 17 hours, filtered again and freeze-dried.

Bone collagen samples (500 µg) were combusted using an Elemental Analyser Flash 2000, coupled with a Delta V Advantage (Thermo Scientific) isotope ratio mass spectrometer for δ^13^C and δ^15^N analysis. Analytical error was estimated to be 0.34 ‰ for δ^13^C and 0.02 ‰ for δ^15^N, based on replicate analysis of the international standard IAEA 600.

The atomic C:N ratio, the carbon and nitrogen content, as well as the yield of collagen, are indicators that can be used to assess potential diagenetic alterations or contaminations of bone collagen. The atomic C:N ratio of unaltered bone collagen ranges from 2.9 to 3.6 [70]. Fresh bone contains approximately 20–22 wt % collagen, which consists of 34 to 43% of carbon and 11 to 16% of nitrogen [71]. The δ^13^C_col_ and δ^15^N_col_ values remain constant until bone collagen yields fall to less than 1% of the total bone mass [72]. The percentage of carbon and nitrogen in unaltered bone collagen should exceed 13% and 4.8%, respectively [73]. Structural carbonate of bone apatite is sensitive to diagenetic alteration [74]. Unlike for bone collagen, there is no well-established indicator to assess the preservation of structural carbonate in bone and enamel apatite [75]. In order to check the preservation of δ^13^C and δ^18^O for bone values, they can be compared with the bone collagen yields to assess the influence of collagen loss on structural carbonate. δ^13^C values of bone structural carbonates can also be compared to those of bone collagen. In herbivores, the spacing (Δδ^13^C_bone-col_) between the two tissues is 7.6±0.5 ‰ [76]. Enamel is considered to be highly resistant to diagenetic alterations and is generally thought to preserve its original δ^18^O_enamel_ and δ^13^C_enamel_ values [77], [78].

### Diet and Mobility through the Analysis of δ^13^C and δ^15^N Values

Measurements of δ^13^C and δ^15^N values are used for investigating past diet because 1) there is a direct relationship between the values of an animal’s tissues and that of its food [79]–[82] and 2) certain food categories present distinctive isotopic values. The δ^13^C values of bone collagen (δ^13^C_col_) are generally 5‰ higher compared to those of consumed plants [81], [83]. However, this fractionation value can vary as carbon collagen is mainly derived from dietary proteins (75% [83]). In contrast, δ^13^C_bone_ or δ^13^C_enamel_ values represent an average carbon input into the whole diet [82], [84]–[85]. Therefore, the combined analysis of collagen and structural carbonate provides complementary dietary data [86]. Different fractionation values between structural carbonate and diet have been determined in the literature [84]–[87]. We used a value of ∼+14 ‰, estimated for ruminant methanogenic herbivores [63], [65], [87]–[88]. The same fractionation value is often used for structural carbonate from both bone and enamel [86]. However, analysis of paired enamel and bone samples for pigs raised on controlled diets showed that enamel apatite was consistently enriched in ^13^C (2–3 ‰) over bone apatite [86]. Because the two tissues formed concomitantly, without seasonal or ontogenetic variations in diet, the difference in tissue-diet spacing appears inherent to the two tissues. This difference was taken into account for camelid palaeodietary reconstructions.

Differences in the fixation of carbon during photosynthesis result in very different δ^13^C values between plants using the C_3_ (Calvin) and C_4_ (Hatch-Slack) photosynthetic pathways [89] at the base of terrestrial food webs. CAM plants that use a third photosynthetic pathway (Crassulacean Acid Metabolism) have intermediate δ^13^C values [90]. Marine plants mostly use the C_3_ pathways but rely on dissolved inorganic instead of atmospheric CO_2_ and also have intermediate δ^13^C values. Many factors can affect the δ^13^C value of terrestrial plants, such as the canopy effect, water availability and soil salinity (reviewed by Szpak et al. [23] for northern Peru). In the Andes, both C_3_ and C_4_ plants are present but C_3_ plants are predominant. A few studies have documented the isotope composition of a variety of terrestrial and marine plants in the Central Andes [23], [91], [92]. Szpak et al.’s [21] comprehensive data provide the most relevant isotopic baseline for the present study, as it was conducted along an altitudinal transect of the Moche Valley. Mean δ^13^C values are –27.6±1.9 ‰ and –13.5±1.0‰ for wild C_3_ and C_4_ plants, respectively. Maize is one of the key products in the diet of farming people and is the only major C_4_ cultivar in the Central Andes. Consumable maize parts exhibited a mean δ^13^C value of –11.8‰ ±0.4 ‰, while leaf mean value was –12.9±0.4 ‰ [23].

Among terrestrial plants only plants that fix atmospheric nitrogen, such as legumes, present clearly distinctive δ^15^N values [93]. Variations in terrestrial plants can be linked to the application of fertilizers for agriculture [23], [94], as well as consequences of local environmental factors, such as aridity and salinity [95], [96]. *Lomas* vegetation in highly arid and saline soil conditions presents a wide range of δ^15^N values and a tendency towards relatively high values [34], [97]. It is usually generally assumed that marine plants have significantly higher δ^15^N values than terrestrial plants but no difference was found between macroalgae and coastal terrestrial plants for northern Peru [23]. δ^15^N analysis has proven fruitful for investigating the food chain level and degree of carnivory in past human diets because of the step-wise enrichment between the consumer’s tissues and its diet [80], [93], [98] and the importance of marine protein because marine food chains are usually longer than terrestrial ones, resulting in higher values for secondary consumers [99], [100]. δ^15^N_col_ values can be affected by many factors, such as pregnancy [101], breastfeeding [102], growth [103], disease [104] or starvation [105].

The existence of geographic variations in isotopic values potentially enables us to distinguish whether camelids found in coastal sites were raised locally or in the highlands. Natural variations in δ^13^C and δ^15^N among camelids bred at different altitudes are expected, with higher δ^13^C and δ^15^N values for lowland than highland camelids (Fig. 2). A positive relationship between the δ^13^C_col_ values and elevation was observed for Argentinian llamas fed on natural pastures at different altitudes (3500–4000 masl) [106] but the reason for this relation – difference in vegetation assemblages or difference in δ^13^C linked to altitude – is not clear. In contrast with this negative relationship between vegetation δ^13^C values and elevation, a positive correlation was found in northern Peru [23] and northern Chile [91]. However, the frequency of C_4_ plants increases with the decrease of altitude and the *puna* is nearly devoid of C_4_ plants. Besides, the upper limit for maize cultivation ends at about 3,500 masl and *puna* crops are limited to C_3_ plant species. The altitudinal distribution of C_4_ plants is linked to temperature, sunlight and precipitation. Thus, the highlands can be characterized as an exclusively C_3_ plant habitat type and camelids are expected to present a pure C_3_ plant diet. Camelids grazing at low elevations have increased access to wild C_4_ plants or could have been fed C_4_ crops. C_4_ plant and mixed C_3_/C_4_ plant diets thus indicate that camelids were raised in the lowlands or intermediate lands. Nevertheless, because the many C_3_ plants that grow on the coast could have potentially provided fodder – various non-maize crops, pods or leaves –, a camelid with a pure C_3_ diet did not necessarily originate from the highlands. Finally, potential access to marine resources in the lowlands could also influence camelid δ^13^C values.

Mean δ^13^C_col_ values of –19.2±0.2 ‰ were measured for domestic camelids in southern Peru [34], [93], [107], while mean δ^13^C_bone_ values of –12.0±0.4 ‰ were measured at La Raya (Peru) [93]. These values – as all following modern animal and plant δ^13^C values –were corrected by 1.5 ‰ to adjust for the Suess effect [108]. There are no available data for modern camelids feeding on natural forage at coastal or very low elevations (<1000 masl), due to the scarcity of wild or domestic camelids in these habitats today. The δ^13^C values of pure C_3_ and pure C_4_-feeders can be predicted from the δ^13^C values of pre-industrial C_3_ and C_4_ plants and used to determine the proportions of C_3_ and C_4_ in the diet of archaeological specimens. The mean δ^13^C for pre-industrial C_3_ and C_4_ plants is estimated to be ∼–26 ‰ by correcting the average value of modern plants for fossil fuel effect (∼1.5 ‰). Szpak et al. [23] suggested an average maize δ^13^C value of –10.3 ‰ to be appropriate for human palaeodietary models in the Central Andes. However, kernels are consumed by humans whereas the leaf was more likely to be consumed by camelids. A mean leaf value of –11.4 ‰, which is similar to the average values of wild C_4_ plants, thus appears more appropriate for camelid dietary studies. Using the +14 ‰ bioapatite-diet spacing and a collagen-diet spacing of 5 ‰, a C_3_ plant-based diet therefore results in an average δ^13^C_enamel_ of ∼–12 ‰, δ^13^C_bone_ of ∼–14 ‰ and a δ^13^C_col_ of ∼–21‰. The mean δ^13^C of pre-industrial C_4_ plants is estimated to be ∼–11.5 ‰ and a pure C_4_-diet should result in an average δ^13^C_enamel_ of∼ +2.5 ‰, δ^13^C_bonel_ of 0 ‰ and a δ^13^C_col_ of –6.5 ‰. The estimated δ^13^C values of pure C_3_ and pure C_4_-feeders were used as end-members in a simple mixing model for calculating the proportion of C_4_ in the diet of archaeological camelids (Tables 1–2). However, absolute values for reconstructed C_4_ proportion values of should be considered with caution because the range of variation in δ^13^C values in natural C_3_ plants is wide (∼18 ‰).

Higher δ^15^N values are expected for domestic camelids feeding extensively on coastal or low-altitude forage, due to the consumption of terrestrial plants with relatively high δ^15^N values and the potential intake of marine resources [100], [109] (Fig. 2). Szpak et al. [23] showed that the foliar δ^15^N values were negatively correlated with altitude and mean annual precipitation along an altitudinal transect of the Moche Valley. The magnitude of difference between animals feeding on wild plants at lower (and drier) altitudes and animals feeding at higher (and wetter) altitudes has been estimated to be 4–6 ‰ [23]. However, the consumption of cultivated plants dependent on irrigation at lower altitudes could partly confound this difference [23]. As mentioned previously, there are no modern data for camelids feeding on natural forage at coastal or very low elevations. Camelids from the coastal site of La Paloma [109] are thought to be wild guanacos and therefore are likely to have consumed natural forage [34]. They present slightly to considerably elevated δ^15^N_col_ values compared to modern *puna* camelids, suggesting that plants characterized by high δ^15^N_col_ values can be naturally selected by wild camelids.

### Origin and Mobility through the Analysis of Structural Carbonate δ^18^O Values

The δ^18^O values of structural carbonate in bone and tooth enamel reflect the δ^18^O value of body water at body temperature. Body water oxygen comes mostly from drinking water and from food, and to a lesser extent from the atmosphere via inhalation, with a predictable fractionation [110]–[112]. Different equations that relate the δ^18^O values of ingested water (δ^18^O_d w_) to the δ^18^O values of structural carbonate and phosphate phases of enamel have been developed in the literature. In this study, we used two equations (Eq. 1) [113] and (Eq. 2) [114] developed for all mammals:
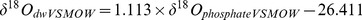
(1)

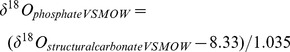
(2)


Basic Andean environmental trends do not seem to have changed over the past 2,000 years (see [11]). δ^18^O_bone_ and δ^18^O_enamel_ values measured in pre-Hispanic remains can be compared to those of modern δ^18^O values for environmental water (δ^18^O_e w_) for assessing an animal’s movement to different geographical areas and estimating the place of residence [115], [116]. Because variations in δ^18^O values of meteoric water (δ^18^O_m w_) are theoretically transferred and retained in the body of living organisms, studies of modern precipitation provide some of the necessary baseline data for residential mobility studies [36]. δ^18^O_m w_ values are governed by the amount of rainfall, altitude, latitude, distance from the coast and temperature [117]–[119], through the preferential loss of ^16^O during evaporation and the progressive loss of ^18^O during precipitation as air masses move inland and upwards [118], [120], [121]. The wide amplitude of temperature, altitude, rate of precipitation and hydrological systems between the different ecological zones of the Andes leads to high natural variations in δ^18^O_m w_ values, which tend to vary predictably across a landmass such as the Andean region in relation to local topography [11] (Fig. 2). The availability of measured δ^18^O_m w_ is limited in the Central Andean region. Preliminary data obtained on an altitudinal transect along the Moche Valley indicate a difference as high as 14 ‰ between the coast and the highlands (4,100 masl) (*Elise Dufour, unpublished data*). δ^18^O_m w_ values for a specific geographical location can be estimated using the Online Isotopes in Precipitation Calculator (OPIC Version 2.2: http://www.waterisotopes.org/). This calculator takes into account the geographic location (latitude, longitude, distance to the sea and altitude) to model the annual and monthly variations in δ^18^O_m w_ for the entire globe [119], [122]. These average values do not take into account the inter-annual variations, the accuracy of which in a given geographical area is linked to the availability of actually measured δ^18^O_m w_ values, which are limited in the case of the Andes. OPIC predicted mean annual δ^18^O_m w_ values are –11.8 ‰ (annual range: –17.3 to –7.0 ‰ SMOW) and –12.8 ‰ (annual range: –17.4 to –8.3 ‰ SMOW) for the *puna* of Tocra and Quiruvilca, respectively, and –4.8 ‰ (annual range: –8.6 to –2.7 ‰ SMOW) for the coastal region of the modern city of Trujillo (−7°99′S).

However, domestic animals (and humans) do not ingest meteoric water but surface waters, or groundwater from wells. Environmental water comprises surface waters (rivers, lakes, reservoirs, irrigation channels) and groundwater. The δ^18^O values of environmental water (δ^18^O_e w_) are not solely tied to rainfall values, which complicates their use [11]. Different processes may result in magnitudes of variation in δ^18^O_e w_ values within regions that may in fact exceed differences between regions [11]. Surface water values are influenced by evaporation, movements between ecozones, mixing with groundwater, El Niño Southern Oscillation events and local topographic relief [24], [123]. Overall, δ^18^O_e w_ values in the Central Andes have been reported to range from –6 to –3 ‰ on the coast, from –9 to –5 ‰ in the *yunga* (intermediate lands) and from –18 to –11 ‰ in the *puna* (data from IAEA/WMO [124] and [11]). In addition, consumption patterns, such as the intake of water collected in cisterns and water exposed to evaporative processes, may also influence δ^18^O_bone_ and δ^18^O_enamel_ values. Finally, weaning has been shown to cause a trophic enrichment of ^18^O in human tissues formed during infancy, due to the equilibration with maternal body water during breastfeeding [125], [126]. Breastfeeding usually lasts for six months in camelids and can influence teeth (such as the first permanent molar) and tissues that form during the first months of life. However, this influence might not be very significant for adult individuals because in most cases the upper part of the M1 is missing due to tooth wear.

**Figure 5:**
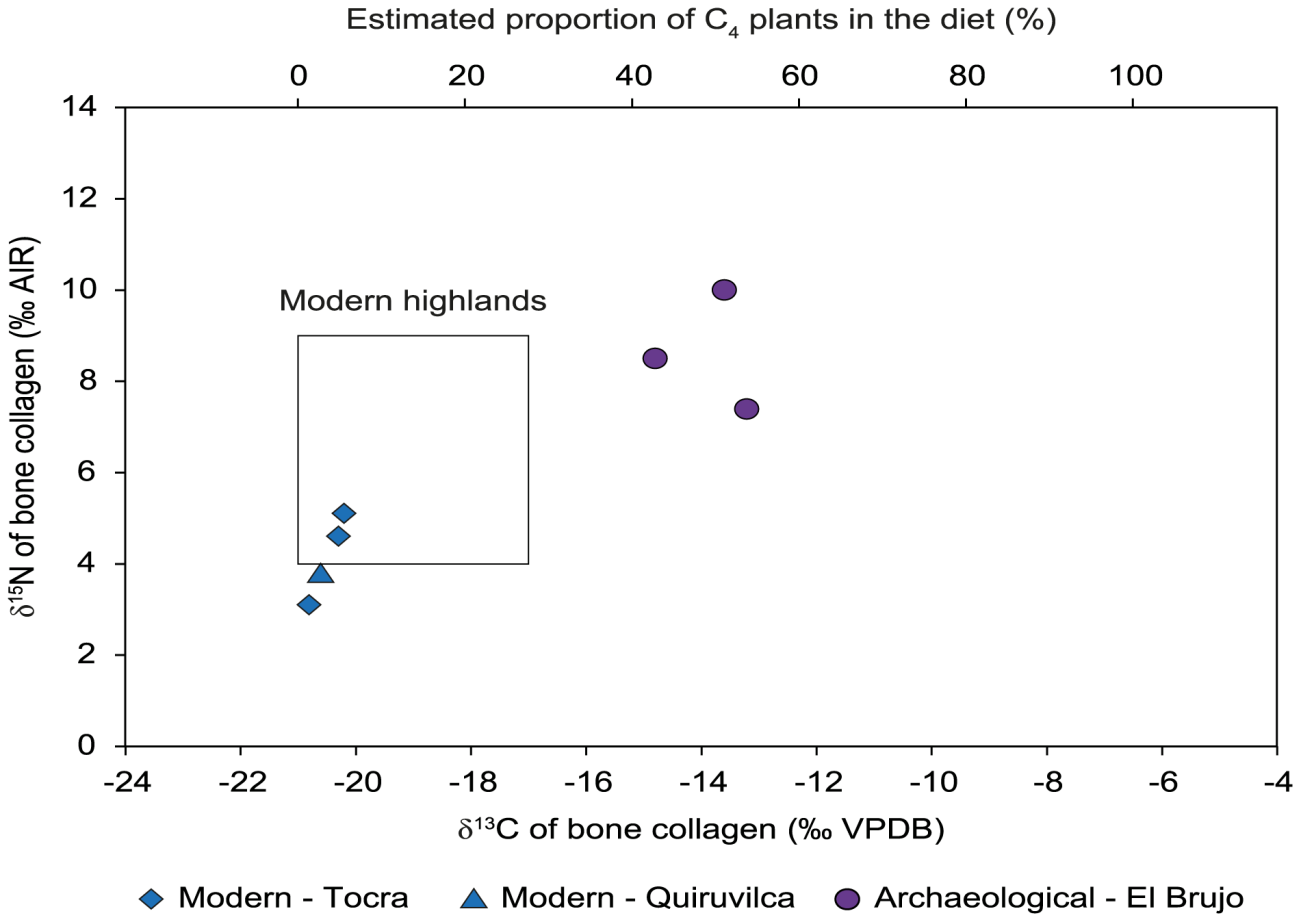
Isotopic composition of bone collagen. Plot of bone collagen δ^13^C and δ^15^N values of archaeological (El Brujo) and modern highland domestic camelids from Tocra and Quiruvilca, compared to the range of variations (represented by dashed lines) of modern specimens, compiled by Thornton et al [34]. The proportion of C_4_ in the diet was reconstructed using mean estimated pre-industrial δ^13^C values of pure-C_3_ and pure-C_4_ feeders.

**Figure 6:**
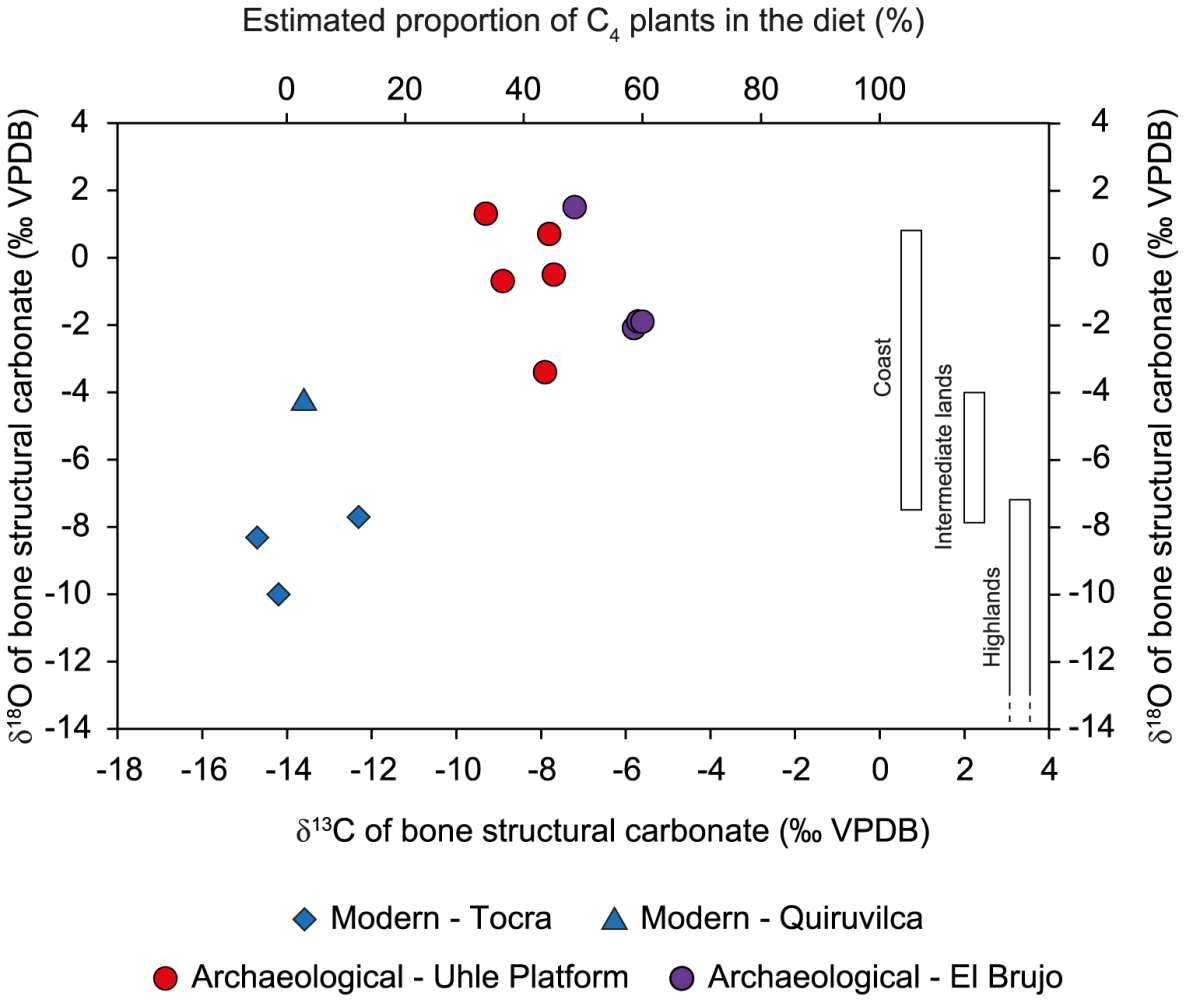
Isotopic composition of bone structural carbonate. Plot of δ^13^C and δ^18^O values (‰ VPDB) of bone structural carbonate of archaeological and modern highland domestic camelids. The proportion of C_4_ in the diet was reconstructed using estimated mean pre-industrial δ^13^C values of pure-C_3_ and pure-C_4_ feeders. Estimated ranges of δ^18^O values for animals raised in the three ecozones are represented by rectangles.

Because of topographic variations in δ^18^O_e w_ and δ^18^O_m w_ values, higher aridity and evaporation of surface water, camelids raised on the coast or at low altitudes should present higher δ^18^O_bone_ and δ^18^O_enamel_ values than camelids raised in the highlands. Based on Eq. 1 and Eq. 2 and OPIC intra-annual variation in δ^18^O_m w_ and IAEA/WMO δ^18^O_e w_ values, δ^18^O_enamel_ of animals living in the lowlands, *yunga* and *puna* can be predicted to vary from –5.8 to +0.5 ‰, –6.2 to –2.3 ‰ and –15 to –5.5 ‰, respectively. The enrichment of structural carbonate in enamel compared to that of bone structural carbonate has been estimated to be 1.7 ‰ in oxygen in pigs [86]. A correction of –1.5 ‰ was then applied to previous estimated values of δ^18^O_enamel_ to estimate δ^18^O_bone_ values for animals living along the coast and in the *yunga* (Figs. 5–6).

## Results

All δ^13^C values of modern specimens presented in Tables 1 and 2 have been corrected by +1.5 ‰ to account for the atmospheric enrichment in ^12^C, caused by the burning of fossil fuel [108], and for comparison with δ^13^C values of archaeological specimens.

### Bone Collagen

Only three on the ten archaeological specimens, all from El Brujo, provided collagen (Table 1, Fig. 5). The bone collagen yields varied from 1.9 to 21.7% for both modern and archaeological specimens. C:N ratio varied from 3.1 to 3.3 for all specimens while the carbon and nitrogen concentrations in bone collagen ranged from 41.2 to 44.1% and from 14.9 to 16.4%, respectively. These criteria were used to check the quality of bone collagen preservation for the El Brujo specimens and showed that δ^13^C_col_ and δ^15^N_col_ values can be used for dietary reconstruction. δ^13^C_col_ and δ^15^N_col_ values for modern specimens varied from –20.8 to –20.2 ‰ and from 3.1 to 5.1‰, respectively (Table 1, Fig. 5). For archaeological specimens, δ^13^C_col_ and δ^15^N_col_ values were higher than those of modern specimens and varied from –14.8 and –13.2 ‰ and from 7.4 to 10.0 ‰, respectively.

### Bone Structural Carbonate

δ^13^C_bone_ and δ^18^O_bone_ values of modern specimens varied from –14.7 and –12.3 ‰ and from –10.0 to –4.2‰, respectively (Table 1, Fig. 6). Both δ^13^C_bone_ and δ^18^O_bone_ values for archaeological specimens were higher than those of modern specimens. δ^13^C_bone_ values varied from –9.3 to –7.4 ‰ and from –7.2 to –5.6 ‰ for Uhle Platform camelids and for El Brujo, respectively. δ^18^O_bone_ values varied from –3.4 to 1.3 ‰ and from –2.1 to 1.5 ‰ for Uhle Platform and El Brujo, respectively (Table 2, Fig. 6). Δδ^13^C_bone-col_ values varied from 7.6 to 7.9 ‰ and were typical of herbivores (Table 2). There was no relationship between variation in δ^13^C_bone_ and δ^18^O_bone_ values and extraction yield indicative of marked diagenetic alteration of bone.

### Enamel Structural Carbonate

**Figure 7:**
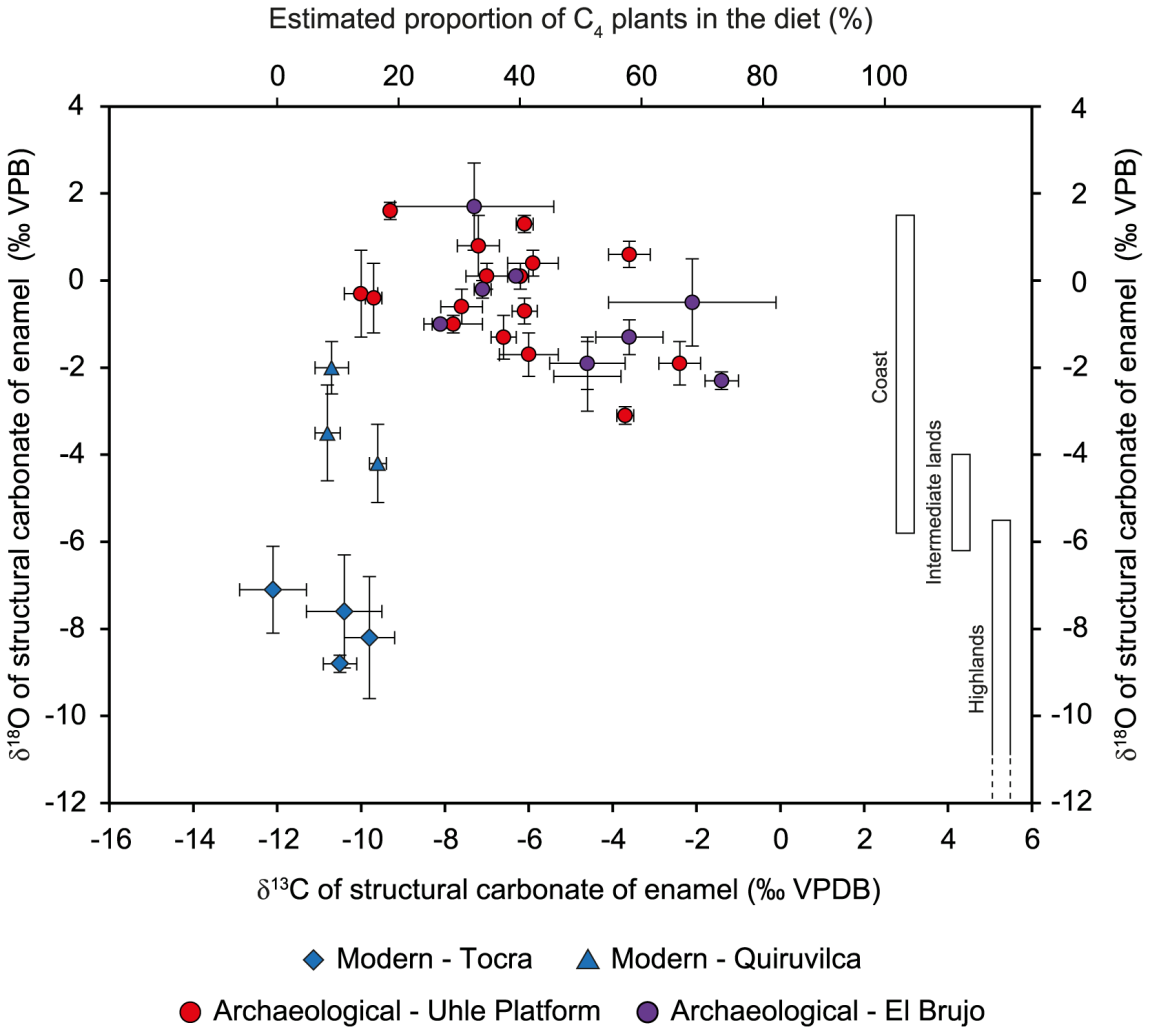
Mean isotopic composition of enamel structural carbonate. Plot of mean δ^13^C and δ^18^O values (‰ VPDB) of enamel structural carbonate for all teeth (molars) of archaeological and modern highland domestic camelids. The proportion of C_4_ in the diet was reconstructed using estimated mean pre-industrial δ^13^C values of pure-C_3_ and pure-C_4_ feeders. Estimated ranges of δ^18^O values for animals raised in the three ecozones are represented by rectangles.

**Figure 8 pone-0087559-g008:**
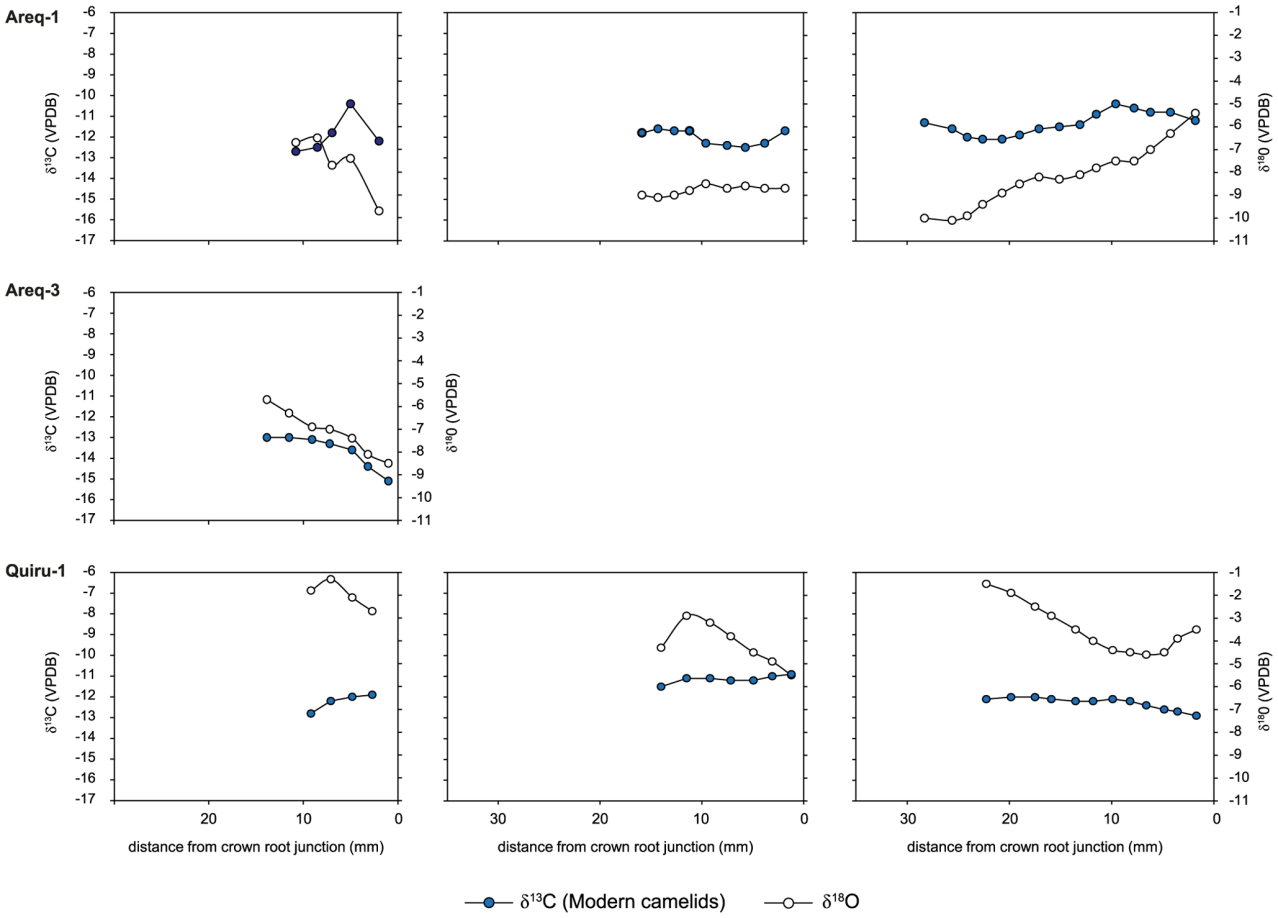
Intra-tooth isotopic composition of enamel structural carbonate of modern camelids. Intra-tooth variations in δ^13^C and δ^18^O values (‰ VPDB) measured along the molar crowns of modern highland domestic camelids and estimation of the contribution of C_3_ and C_4_ plants during crown growth. For each specimen, the left graph is for the M1 molar (except for Areq-3), the middle graph for the M2 molar and the right graph for the M3 molar. On each graph, the left axis refers to δ^13^C_enamel_ values and the right to δ^18^O_enamel_ values.

**Figure 9:**
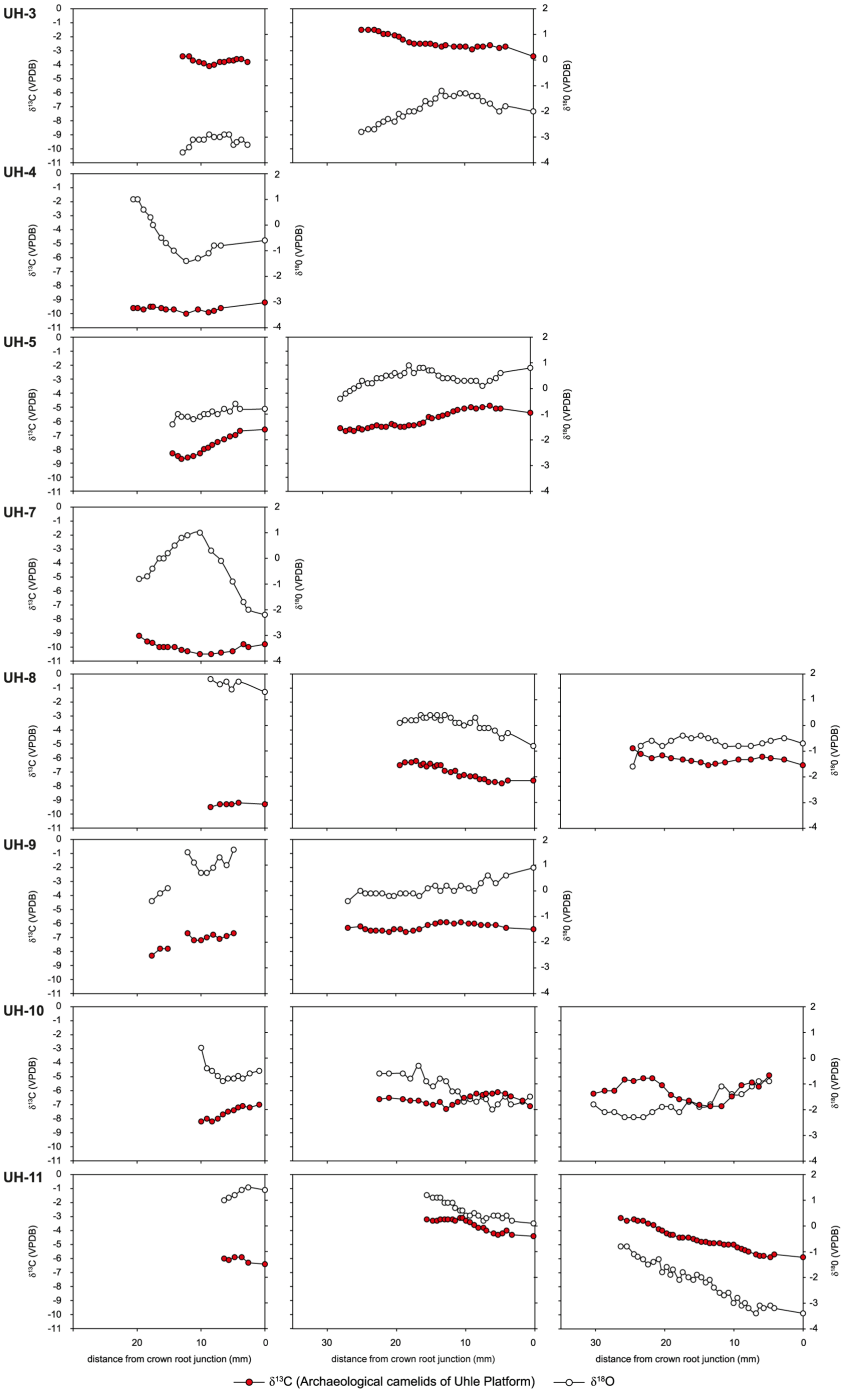
Intra-tooth isotopic composition of enamel structural carbonate of Uhle Platform camelids. Intra-tooth variations in δ^13^C and δ^18^O values (‰ VPDB) measured along the molar crowns of archaeological and modern highland domestic camelids and estimation of the contribution of C_3_ and C_4_ plants during crown growth. For each specimen, the left graph is for the M1 molar, the middle graph for the M2 molar and the right graph for the M3 molar. On each graph, the left axis refers to δ^13^C_enamel_ values and the right to δ^18^O_enamel_ values.

**Figure 10:**
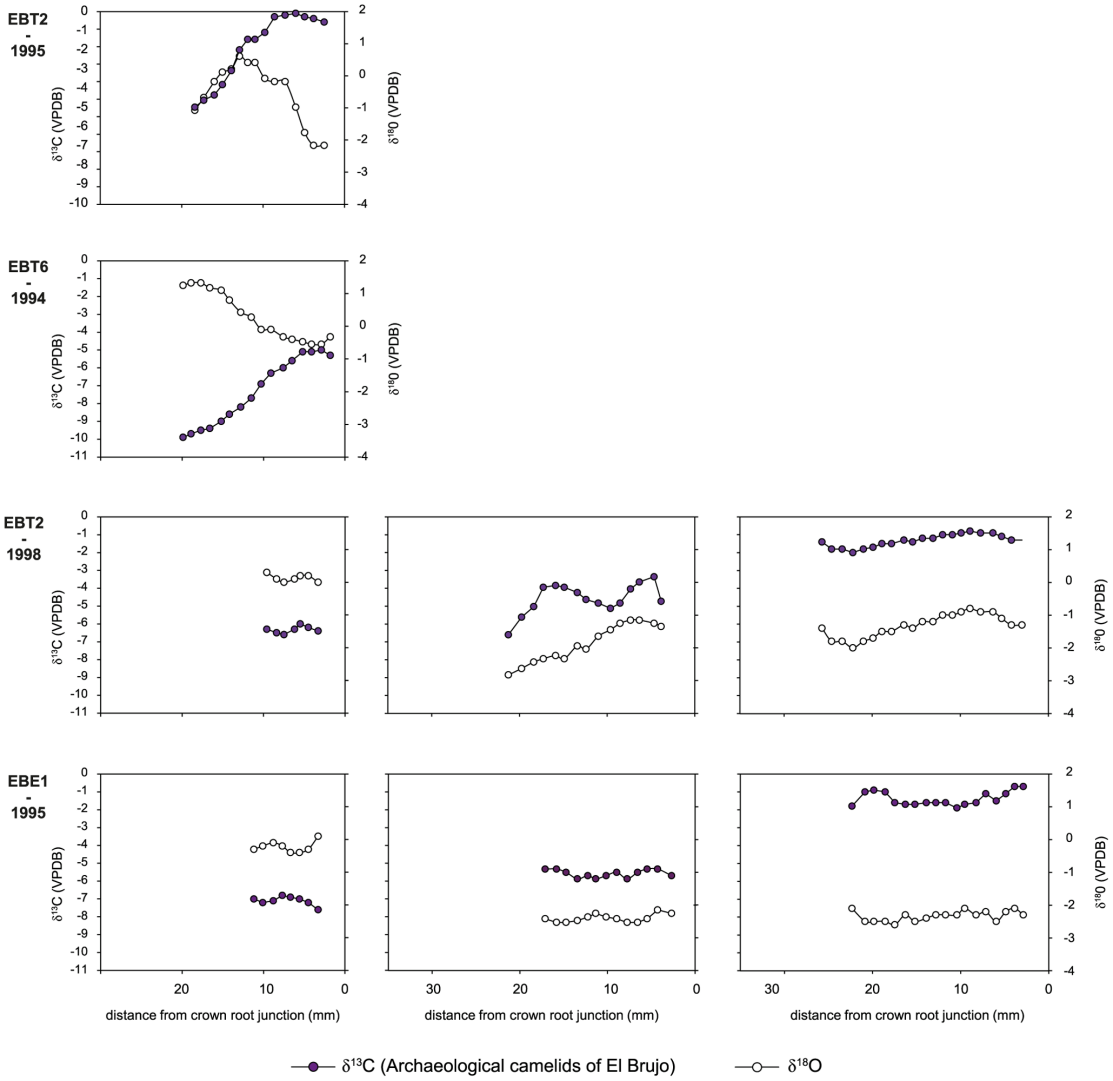
Intra-tooth isotopic composition of enamel structural carbonate of El Brujo camelids. Intra-tooth variations in δ^13^C and δ^18^O values (‰ VPDB) measured along the molar crowns of archaeological and modern highland domestic camelids and estimation of the contribution of C_3_ and C_4_ plants during crown growth. For each specimen, the left graph is for the M1 molar, the middle graph for the M2 molar and the right graph for the M3 molar. On each graph, the left axis refers to δ^13^C_enamel_ values and the right to δ^18^O_enamel_ values.

For modern camelids, δ^13^C_enamel_ values ranged from –13.6 ‰ (Areq-3) to –8.9 ‰ (Areq-1), with mean tooth values ranging from –12.1±0.8 to –9.6±0.2 ‰ (Table 3, Figs. 7–8). Intra-tooth variation ranged from 0.6 (M2 of Quiru-1) to 2.3‰ (M1 of Areq-1), while intra-individual variations (for specimens with three sampled teeth) were 2.0 ‰ for Quiru-1 and 2.3 ‰ for Areq-1. Archaeological specimens usually present higher δ^13^C_enamel_ values than modern specimens (Table 3, Figs. 7–9–10). For Uhle Platform, δ^13^C_enamel_ values varied from –10.5 to –1.5 ‰, with mean tooth values ranging from –10.0±0.4 to –2.4±0.5 ‰. Intra-tooth variation ranged from 0.3 (M1 of UH-8) to 2.8 (M3 of UH-11). Maximum intra-individual variation is 3.3 ‰ for the three molars of UH-8. At El Brujo, δ^13^C_en_ values varied from –9.9 to –0.1 ‰ with mean tooth values varying from –8.1±0.2 to –1.4±0.4 ‰ (EBE1-1995). Intra-tooth variation ranged from 0.6 to 5.4 ‰ (EBT2-1995) while intra-individual variation was 4.7 ‰ for EBT2-1998 and 6.9 ‰ for EBE1-1995.

Modern camelid δ^18^O_enamel_ values ranged from –10.1 (Areq-1) to –1.3 ‰ (Quiru-1), with mean tooth values ranging from –8.8±0.2 to –2.0±0.6 ‰ (Table 3, Fig. 7–8). Intra-tooth variation ranged from 1.4 to 4.7 ‰. Archaeological specimens usually presented higher δ^18^O_enamel_ values than the modern ones but values overlapped (Table 3, Figs. 7–9–10). Uhle Platform values varied from –3.6 ‰ (UH-3) to 1.8‰ (UH-8). Intra-tooth maximum variation ranged from 0.2 to 2.6 ‰. At El Brujo, δ^18^O_enamel_ values ranged from –2.9 ‰ (EBT2-1995) to 3.1 ‰ (EBT6-1994). Intra-tooth variation ranged from 0.3 to 2.8 ‰.

The M3 from modern specimens presented cyclic variations in δ^18^O_enamel_ values (Fig. 8) whereas no cyclic variation was observed for the M3 of archaeological specimens (Figs. 9–10). Only the youngest individuals (M1 of UH-4, UH-7, EBT2-1995, EBT6-1994) exhibited cyclicity in δ^13^C_enamel_ and δ^18^O_enamel_ (Figs. 9–10).

## Discussion

In the present study, the life history of Mochica archaeological camelids was reconstructed through the analysis of the stable isotope composition of bone and enamel and comparisons with modern highland (*puna*) specimens. Archaeological camelids are discriminated from modern specimens by δ^13^C values measured in both organic and inorganic bone fractions. δ^13^C_col_ and δ^13^C_bone_ values of modern specimens from the *puna* of Tocra (Areq-1, Areq-2, Areq-3) and Quiruvilca (Quiru-1) were consistent with predicted and previously measured values from the literature and all indicate the sole consumption of C_3_ plants (Figs. 5–6). Considering that archaeological camelid diets consisted exclusively of C_3_ and C_4_ plants, the reconstructed proportion of C_4_ plants is consistent among specimens, varying from 43 to 54% and from 34 to 60%, based on bone collagen and bone structural carbonate analysis respectively (Table 2, Figs. 5–6). δ^18^O_bone_ values for archaeological specimens fall within the predicted range for animals raised in the lowlands and are distinct from those from modern specimens (Fig. 6). However, while the three specimens from Tocra fall within the predicted range of variation for animals raised in the *puna*, δ^18^O_bone_ values of the Quiruvilca alpaca (Quiru-1) were higher.

δ^13^C_col_ or δ^13^C_bone_ and δ^15^N_col_ values point to a mixed C_3_/C_4_ diet and relatively high δ^18^O_bone_ values indicate an arid habitat, suggesting long-term herding of Mochica camelids outside the *puna* C_3_ pastures. This lowland presence of Mochica domestic camelids is in contradiction with the modern distribution of camelids and the classical view that considers it impossible to maintain herds outside the *puna* grasslands. However, it does not necessarily challenge the model of verticality and exchange between the ecological zones of Andean pastoralism. Adult camelids analysed in the present paper could have spent their youth in the highlands and then been brought to the coast as part of a caravan. Their long-term, mixed C_3_-C_4_ diet signal, associated with an aridity signal, would thus have resulted from a diachronic switch in diet associated with the change in geographic location. Because of bone remodeling, the isotopic signal resulting from this diachronic switch cannot be distinguished from that of an entire life spent on the coast feeding on a mixed C_3_-C_4_ diet.

**Figure 11:**
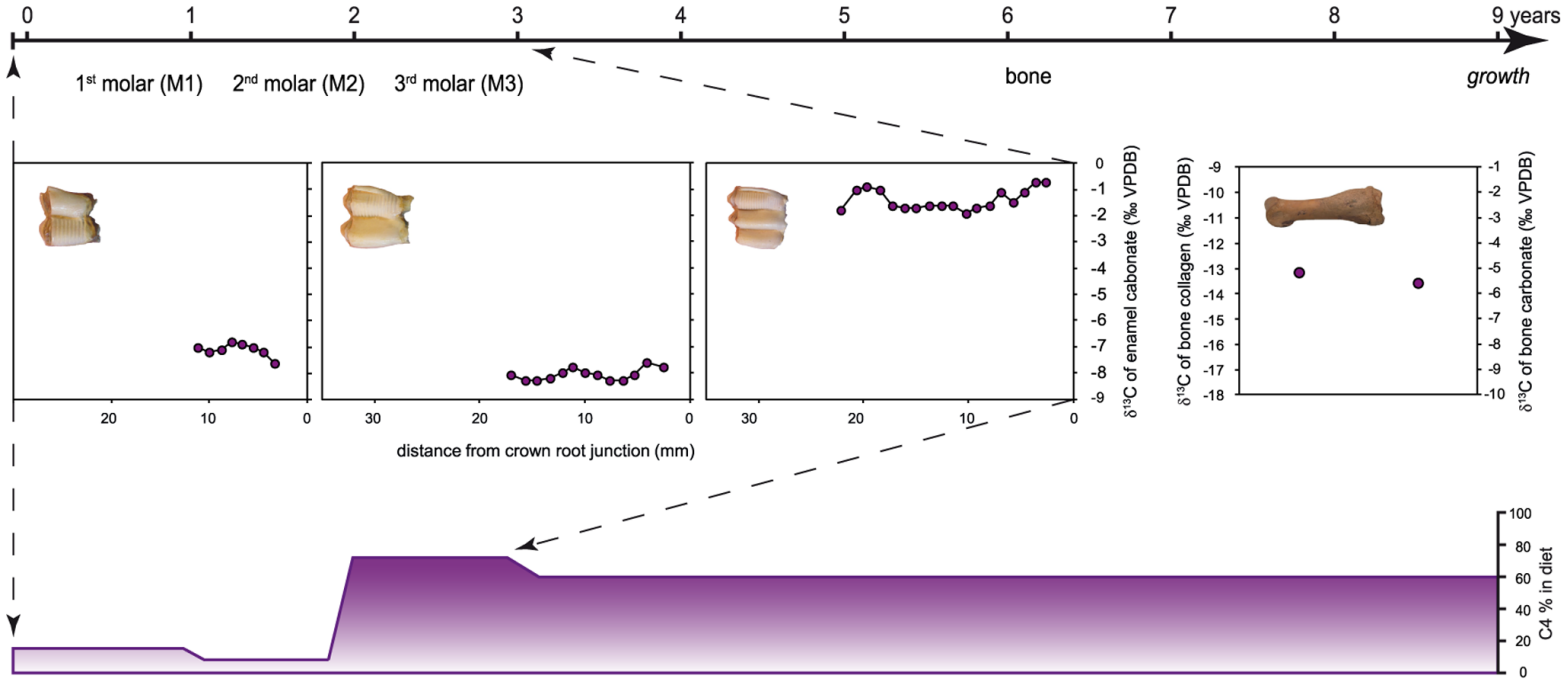
Reconstruction of diachronic changes in the contribution of C_4_ plants to camelid diet. Dietary contribution of C_4_ plants over the first years of life of specimen EBE1-1995 and in the long term is estimated by the combined analysis of enamel (δ^13^C_enamel_) from the three molars and the bulk analysis of collagen (δ^13^C_col_) and structural carbonate (δ^13^C_bone_) from bone.

The analysis of tooth enamel reveals more diverse and complex life histories for Mochica camelids than bone analysis. Whereas all the modern specimens had a pure C_3_ diet during their youth, most of the archaeological camelids consumed C_4_ plants. The mean C_4_ contribution to the juveniles’ diet was much more variable than the long-term C_4_ contribution recorded in bone, ranging from 14 to 73% (Table 3; Fig. 7). Although the use of the mixing model leads to the reconstruction of a small proportion of C_4_ for the M1 from the two youngest camelids from the Uhle Platform (UH-4 and UH-7) and from an adult (UH-8), their δ^13^C_enamel_ values were very similar to those of modern *puna* camelids (Table 3, Fig. 6). Keeping in mind the high variability in δ^13^C values for C_3_ plants, these three animals may have had a pure C_3_ diet throughout their first year of life. Considering the other specimens, diet change during youth was usually moderate (10–25%), except for one adult from El Brujo (EBE1-1995), for which the C_4_ contribution increased by 50% between the second and third years of life (Table 3, Fig. 11). The δ^18^O_enamel_ values for modern animals from Tocra fitted into the estimated range for *puna* camelids, while the alpaca (Quiru-1) from Quiruvilca exhibited higher δ^18^O_enamel_ values than predicted values (Table 2, Fig. 7). In the absence of a comprehensive survey of δ^18^O values of surface water ingested by camelids, the reason for this discrepancy remains unknown. δ^18^O_enamel_ values of archaeological specimens partially overlap those of modern camelids. However, they all fitted into the estimated range of variation for the lowlands or intermediate lands (Fig. 7), suggesting a youth spent in environments characterized by high δ^18^O_e w_ values or surface water affected by a high evaporation rate. Most individuals exhibited moderate variations in δ^18^O_enamel_ values during youth, suggesting no major change in habitat, except for one adult from the Uhle Platform (UH-11) that might have moved to a habitat characterized by lower δ^18^O_e w_ values or less arid conditions, probably at a higher altitude.

The comparison of the enamel isotopic signal with that of bone indicated that most adults were kept outside the *puna* all their life, as shown by the incorporation of C_4_ plants. However, the comparison between these two tissues also identified an ontogenetic switch in diet and possible residential mobility throughout life for three animals from the Uhle Platform (UH-4, UH-7 and UH-8). This ontogenetic switch indicates a pure (or almost pure) C_3_ diet during the first year of life, followed by a mixed C_3_-C_4_ plant diet afterwards. A pure C_3_ diet alone is not indicative of the animal’s geographic origin because C_3_ plants dominate in all habitats. Many C_3_ species that thrive on the coast could have potentially provided fodder – various non-maize crops, shrub leaves or pods − for camelids. *Prosopis* sp. remains have been reported from camelid coprolites dated of the Chimú Period [127]. Due to relatively low M1 δ^18^O_enamel_ values for the two youngest camelids (UH-4 and UH-7; Figs. 7,9) – and the wide variation in δ^18^O_enamel_ values for modern *puna* camelids – it is not possible to identify their whereabouts during their first year of growth. Theoretically, they could have been born in the *puna* but could have fed in the lowlands during at least several months of the year, and again before death (because of the mixed C_3_/C_4_ diet recorded by bone collagen). Regarding the adult specimen (UH-8), the arid signal exhibited in the M1 δ^18^O_enamel_ values (the highest values recorded for all Uhle Platform individuals) suggests that this animal was bred in the lowlands where it switched diet during the second year of life (Table 3; Fig. 9).

Despite the importance of domestic camelids in the Mochica culture, management strategies and pastoralism remain poorly documented. In the present study, adults and juveniles of more than 1 year were analysed. Combined isotopic measurements of bone and tooth enamel clearly showed that all the adults and most juveniles spent their all life outside the *puna*, in the lowlands (and/or intermediate lands for UH-11). Furthermore, all juveniles were present locally at least several months before slaughtering. The probable pure C_3_ diet during early life for two young individuals from Uhle Platform (UH-4 and UH-7) raises questions concerning their geographic origin. These individuals could have either been raised locally or in the highlands. If so, they would have subsequently reached the coast during trips for product exchanges (fish, cotton, *aji*, corn, gold, silver, etc.) between ecological zones. Although young individuals are not considered robust enough to carry large burdens during such trips [7], [13], they might have accompanied their mothers. This latter hypothesis cannot be totally excluded, even though ethnographic data indicate that young llamas do not integrate caravans before adulthood, when they are around two years old [21]. We can thus assume that UH-4 and UH-7were born in the lowlands like the other juveniles and the adults.

At both El Brujo and Uhle Platform, animals used for funerary and ritual purposes were kept in the lower part of the valleys. Local herds were maintained both for slaughtering purposes and reproduction, as indicated by demographic data based on the study of archaeozoological remains and domestic camelid ethology [7], [13]. Local breeding not only implies the presence of young age classes outside the *puna* during the Mochica period but also that of gregarious adult females. The latter do not travel during the gestation period, which lasts from eleven to twelve months [9]. Moreover, this suggests that camelid breeding was an important activity. Young camelids were sacrificed before reaching their maximum meat yield by weight and consequently herds had to be large enough to absorb the loss of young immature animals. Herds might have been large or alternatively small but numerous, at the scale of a site or a valley. While large herding has also been suggested by the increased number of camelid remains [16], there is currently no available estimate of herd size during Mochica times.

Camelid management outside the modern geographical range has been documented for middle valley areas in Andean regions where richer pasturelands are available and foddering might have been practiced during pre-Hispanic times [34], [35], [128], [129]. As for the Peruvian coast where arid conditions prevail, questions subsist concerning the availability and precise location of food resources to maintain herds. *Lomas* are present close to Cerro Campana, between the Moche and Chicama valleys, and can be considered as a potential herding location, at least during the austral winter, as suggested for other sites in Peru [34]. In intermediate lands, C_4_ plants are relatively rare and high δ^13^C_col_ (or δ^13^C_bone_) values, such as those recorded at the Middle Horizon Wari-affiliated site of Conchopata [128], can be confidently interpreted as the result of the consumption of maize. On the coast, wild C_4_ species, such as the saltgrass *grama salada* (*Distichlis spicata*), can locally form relatively large patches and pastures where herders could have taken their animals. C_3_ plants nevertheless dominate the Peruvian flora and it could be difficult for herders to find wild C_4_ pastures capable of contributing as much as 50–70% of the diet of some camelids. Maize but also marine algae can supplement the diet of wild or domestic herds [130], [131], at least seasonally when the terrestrial pastures are restricted. Records of this practice during ancient times are hard to come by as seaweed is rarely preserved in archaeological sites. High δ^13^C_col_ and δ^15^N_col_ values measured at the coastal sites of central and south Peru of La Paloma and Chilca were interpreted as lowland breeding, on the arid coast itself [109]. These high values were attributed to the consumption of non-terrestrial resources, such as marine plants or aquatic vegetation growing along lagoons, riverbanks or the shores of saltwater lakes because of assumed distinctive (higher) δ^15^N values for marine plants compared to terrestrial plants [109], [132]. In fact, no distinction between the two types of plants was found along the northern coast of Peru [23] that could also explain the high δ^15^N_col_ values of archaeological camelids. Marine algae have a mean δ^13^C value of –15.0 ‰ [23]. Their contribution to the diet of Mochica camelids should have been as high as 75% to explain the highest measured δ^13^C_col_ and δ^13^C_bone_ values. Although this high proportion of marine algae seems unlikely, they may still have contributed to the camelid diet, along with C_4_ plants. This hypothesis cannot be ruled out by the isotopic tracers used in the present study.

The geographical origin of camelids appears difficult to assess from δ^18^O_enamel_ and δ^18^O_bone_ values, but they might nevertheless provide insights into herding practices. A negative trend in the relationship between δ^13^C_enamel_ and δ^18^O_enamel_ values is observed when all archaeological teeth are considered, suggesting that there is no connection between aridity and dietary signals (Fig. 6). A positive relation was expected because the lower the elevation, the higher the aridity, the δ^18^O_e w_ values and the frequency of wild C_4_ in the vegetation assemblage. The disconnection between aridity and dietary signals could result from the consumption of water and maize (and other cultivated crops) grown at various altitudes from the arid coast to intermediate lands. Cultivation on the coast is rendered possible by the presence of irrigation networks supplied by rivers from the Andes, which have different δ^18^O_e w_ values from those predicted at local altitudes, as well as different precipitation rates and temperatures. The control of drinking by the herders is not only suggested by the absolute δ^18^O_enamel_ values, but also by the difference in the shape of isotopic intra-tooth profiles between archaeological and modern specimens. The two modern adult alpacas (Areq-1 and Quiru-1) were raised on natural *puna* pastures all year around, without fodder, and widescale geographic movement over throughout life. The intra-tooth cyclic variation in δ^18^O_enamel_ recorded in their M3 is linked to seasonal variation in temperature and δ^18^O_e w_ values [36]. In contrast – except for the M1 from the youngest specimens (UH-4, UH-7, EBT6-1994, EBT2-1995) – most archaeological Mochica camelids do not display cyclicity in δ^18^O_enamel_ intra- tooth cyclic variations, independently of the proportion of C_4_ plants in their diet (Fig. 9–10). The flat profiles for the δ^18^O_enamel_ values of Mochica camelids not only suggest that no important change in geographic location occurred (except UH-11), but also that drinking water was not affected by seasonal variations, such as natural surface water and precipitation, but came from controlled sources such as wells or canals.

Combined isotopic measurements of bone and tooth enamel indicate that most Mochica camelids relied upon substantial maize foddering on coastal irrigated (and/or fertilized) fields or at different elevations in the valleys. Maize played a key role in the political economy and has constituted a primary component of the diet of diverse Andean polities and cultures from at least the late Archaic [127], [133]–[134]. In the Moche Valley, the intensification of maize production appeared during the Gallinazo phase [135] and maize was a crucial crop during the development of the Mochica state [127], [136]–[138]. Herders could have let the animals graze on the stalks in maize fields, forage in cultivated plots or fields, or brought stalks to the herds in corrals and pens. Further archaeological research is needed to determine if maize culture and herds were spatially segregated or in close relation with each other. No corrals or pens used for keeping the herds have been found in the proximity of Huacas de Moche and El Brujo. Thus, if the camelids lived on the coast, their settlement was not in the direct vicinity of the sites. Our study showed that the animals were bred in the low and/or middle valleys and that none of the domestic camelids originated from the *puna*. As the Mochica polities exerted their political and social power at the scale of the valleys, the provisioning of domestic camelids might also have been organized at this level. All animals were bred in the lowlands but had different life histories, with the exception of the three specimens (UH-8, UH-9 and UH-10) recovered from the same ceremonial context (Element 21) at the Uhle Platform. They shared similar trends in life histories despite differences in age and may represent a single ritual event (Fig. 9). Part of the inter-individual variability could be linked to the fact that remains were recovered from different chronological phases of occupation. It also suggests that camelids came from various herds presenting difference in herding management strategies. The presence of camelids from different geographic origins (classified as local and non-local) at the same site has previously been related to the different functions of camelids: sacrifice *versus* meat or wool production [34]–[35], [128]. The site of Tiwanaku is situated in a zone of rich *puna* pastures and the provisioning of non-local – but still originating from the highlands – camelids [35] illustrates the complexity of ritual contexts. The present work concerns camelids from funerary and ritual contexts. The lack of analysis of remains from domestic contexts does not enable us to discuss the specialization of Mochica herds for meat procurement or animal sacrifice, as was described for Inka times.

## Conclusion

The present study underlines the adaptive capacities of the Mochica people to their environment. Irrigation allowed for the development of extensive agriculture in a very arid environment. Herders also adapted their practices to this difficult environment. This study also emphasizes the role of maize in different aspects of Mochica economic life. Further work will be conducted on Mochica camelids from different valleys to confirm these first results and to compare herding practices on a regional scale. Strong relationships might have been established between two major religious centers, such as the Huacas de Moche and El Brujo, and their hinterlands. A better understanding of these relationships is required in order to describe how herd management was spatially organized. Camelids from domestic contexts should also be analysed to compare them with camelids from ritual contexts, to determine whether or not they have the same origin and to gain insights into meat exchange in the different Andean ecozones. Our study also indicates that diversity in herding practices existed in the lowlands, as already described for higher altitudes, even though only animals with similar functions (funerary and ritual) were analysed. Species-specific identification of llamas and alpacas was not possible from the osteological study of archaeological remains. This would, however, provide a better understanding of the relation between camelid functions and herding practices.

An increasing number of studies have used stable isotopic analyses to document camelid diet and herding practices in Peru, Chile and Argentina, during the pre-Hispanic period [34], [128]–[129], [139]–[141]. Together with the data presented in this study, these analyses have identified variations in the geographic locations of camelid breeding and pastureland and/or foddering practices outside the modern geographic range. Our work underscores regional and temporal variations in Andean pastoralism and disparity with the classical model of vertical exchange. Most previously published studies were performed on bone tissue and make no reference to the age of the sampled individuals. Because of the remodeling of bone tissue, age is essential for reconstructing the individual life history of each specimen and for identifying the place of birth. The serial analysis of tooth enamel brings new information on camelid pastoralism and complements bone collagen analysis. The combined analysis of different tissues, which form at different times, enables us to detect potential diachronic change in diet and geographical location throughout an animal’s life. Besides, tooth enamel is better preserved over time than bone collagen. The poor quality of preservation of bone collagen in the Moche Valley was also observed for human remains at Cerro Oreja [135]. Moreover, enamel analysis offers the opportunity to independently assess the geographic habitat of organisms through climate records. δ^18^O_enamel_ values provide some information but inferences are limited so far because the environmental and biological factors that might influence δ^18^O_enamel_ values need to be better understood. The influence of the circulation of surface water that might mask the difference in δ^18^O_e w_ values between ecological zones, breastfeeding and milk consumption are some of the factors that have to be documented. The duration of tooth mineralization in domestic camelids would also need to be determined. Finally, a more precise reconstruction of the geographic location of pastureland and mobility is expected from the combination of the measurement of δ^13^C, δ^15^N and δ^18^O_enamel_ values with ^87^Sr/^86^Sr values.

## References

[1] Murra J (1975) El control vertical de un máximo de pisos ecológicos en la economía de las sociedades andinas. In: Murra J, editor. Formaciones económicas del mundo andino. Lima: Instituto de Estudios Peruanos. 59–115.

[2] Bonavia D (1996) Los Camélidos sudamericanos. Una introducción a su estudio. Lima: Ifea-Upch-Conservación International. 845 p.

[3] Flores Ochoa JA, MacQuarrie K, Portus J (1994). Oro de los Andes. Las Llamas, Alpacas, Vicuñas y Guanacos de Sudamérica. Barcelone: Jordi Blassi, 2 volumes.

[4] Browman D (1974) Pastoral nomadism in the Andes. Current Anthropology 15: 188–196.

[5] Nuñez L, Dillehay TS (1978) Movilidad giratoria, armonía social y desarrollo en los Andes Meridionales: Patrones de tráfico e interacciones económicas. Antofagasta, Universidad Católica Del Norte. 170p.

[6] Van Buren M (1996) Rethinking the Vertical Archipelago. American Anthropologist 98: 338–351.

[7] Goepfert N (2012) New zooarchaeological and funerary perspectives on Mochica Culture (100–800 A.D.), Peru. Journal of Field Archaeology 37: 104–120.

[8] Shimada M, Shimada I (1985) Prehistoric llama breeding and herding on the North Coast. American Antiquity 50: 3–26.

[9] Wheeler JC (1991) Origen, evolución y status social. In: Fernandez-Baca S, editor. Avances y perspectivas del conocimiento de los camélidos Sudamericanos. Santiago du Chili: Fao. 11–48.

[10] Franklin WL (1982) Biology, ecology, and relationship to man of the South American camelids. In: Mares MA, Genoways HH, editors. Mammalian biology in South America. Pittsburgh: University of Pittsburgh. 457–489.

[11] Knudson KJ (2009) Oxygen isotope analysis in a land of environmental extremes: the complexities of isotopic work in the Andes. International Journal of Osteoarchaeology 19: 171–191.

[12] Topic TL, McGreevy TH, Topic JR (1987) A comment on the breeding and herding of llamas and alpacas on the north Coast of Peru. American Antiquity 52: 832–835.

[13] Goepfert N (2011) Frayer la route d’un monde inversé. Sacrifice et offrandes animales dans la culture Mochica (100–800 apr. J.-C.), côte nord du Pérou. Oxford: British Archaeological Reports International Series 2278 (BAR). 428 p.

[14] Lozada MC, Buikstra JE, Rakita G, Wheeler JC (2009) Camelid Herders: The forgotten specialists in the coastal Señorío of Chiribaya, southern Perú. In Marcus J, Williams PR, editors. Andean Civilization: A Tribute to Michael E. Moseley. Los Angeles: Cotsen Institute of Archaeology-Ucla. 351–377.

[15] Pozorski SG (1976) Prehistoric subsistence patterns and site economics in the Moche valley, Peru. PhD in Anthropology. Austin, University of Texas. 475 p.

[16] Pozorski SG (1979) Late Prehistoric llama remains from the Moche Valley, Peru. Annals of Carnegie Museum 48: 139–169.

[17] Shimada M, Shimada I (1981) Explotación y manejo de los recursos naturales en Pampa Grande, sitio Moche V. Significado del análisis orgánico. Revista del Museo Nacional 45: 19–74.

[18] Pozorski SG, Pozorski T (1979) An early subsistence exchange system in the Moche Valley, Peru. Journal of Field Archaeology 6: 413–432.

[19] Vásquez Sánchez VF, Rosales Tham TE, Coronado Tello L (2001) Evidencias arqueológicas de crianza de camélidos en los siglos V y VI en la Costa Norte de Perú. In: Mengoni Goñalons GL, Olivera DE, Yacobaccio HD, editors. El Uso de los camélidos a través el tiempo. Buenos Aires: Ediciones del Tridente-GZC-Icaz. 241–260.

[20] Pillsbury J (2001) Moche Art and Archaeology. Washington DC: National Gallery of Art. 344p.

[21] Lecoq P (1987) Caravanes de lamas, sel et échanges dans une communauté de Potosí, en Bolivie. Bulletin de l’Institut Français d’Études Andines 16: 1–38.

[22] Pulgar Vidal J (1981) Geografía del Perú: las ocho regiones naturales del Perú. Lima: Universo. 313p.

[23] Szpak P, White CD, Longstaffe FJ, Millaire JF, Vasquez Sanchez V (2013) carbon and nitrogen isotopic survey of northern Peruvian plants: Baselines for Paleodietary andPaleoecological Studies. Plosone 8: 1–28. 10.1371/journal.pone.0053763 PMC3547067 23341996

[24] Webb EC, White CD, Longstaffe FJ (2013) Exploring geographic origins at Cahuachi using stable isotopic analysis of archaeological human tissues and modern environmental waters. International Journal of Osteoarchaeology 23: 698–715.

[25] Buzon MR, Conlee CA, Bowen GJ (2011) Refining oxygen isotope analysis in the Nasca region of Peru: an investigation of water sources and archaeological samples. International Journal of Osteoarchaeology 21: 446–455.

[26] Falabella F, Planella MT, Tykot RH (2008) El maíz (*Zea mays*) en el mundo Prehispánico de Chile Central. Latin American Antiquity 19: 25–46.

[27] Finucane BC (2009) Maize and Sociopolitical Complexity in the Ayacucho Valley, Peru. Current Anthropology 50: 535–545.

[28] Hastorf C (1990) The effect of the Inka state on Sausa agricultural production and crop consumption. American Antiquity 55: 262–290.

[29] Kellner CM, Schoeninger MJ (2007) A simple carbon isotope model for reconstructing prehistoric human diet. American Journal of Physical Anthropology 133 (4): 1112–1127. 10.1002/ajpa.20618 17530667

[30] Tykot RH, Staller JE (2002) The importance of early maize agriculture in Coastal Ecuador: New data from La Emerenciana. Current Anthropology 43: 666–677.

[31] Knudson KJ, Williams SR, Osborne R, Forgey K, Williams PR (2009) The geographic origins of Nasca trophy heads in the Kroeber collection using strontium, oxygen, and carbon isotope data. Journal of Anthropological Archaeology 28: 244–257.

[32] White CD, Nelson AJ, Longstaffe FJ, Grupe G, Jung A (2009) Landscape bioarchaeology at Pacatnamu, Peru: inferring mobility from δ^13^C and δ^15^N values of hair. Journal of Archaeological Science 36: 1527–1537.

[33] Turner BL, Kamenov GD, Kingston JD, Armelagos GJ (2009) Insights into immigration and social class at Machu Picchu, Peru based on oxygen, strontium, and lead isotopic analysis. Journal of Archaeological Science 36: 317–332.

[34] Thornton EK, DeFrance SD, Krigbaum J, Williams PR (2011) Isotopic evidence for Middle Horizon to 16th Century camelid herding in the Osmore Valley, Peru. International Journal of Osteoarchaeology 21: 544–567.

[35] Knudson KJ, Gardella KR, Yaeger J (2012) Provisioning Inka feasts at Tiwanaku, Bolivia: the geographic origins of camelids in the Pumapunku complex. Journal of Archaeological Science 39: 479–491.

[36] Goepfert N, Dufour E, Gutiérrez B, Chauchat C (2013) Origen geográfico de camélidos en el periodo mochica (100–800 AD) y análisis isotópico secuencial del esmalte dentario: enfoque metodológico y aportes preliminares. Bulletin de l’Institut Français d’Etudes Andines 42: 25–48.

[37] Balasse M, Ambrose SH, Smith AB, Price TD (2002) The seasonal mobility model for prehistoric herders in the south-western Cape of South Africa assessed by isotopic analysis of sheep tooth enamel. Journal of Archaeological Science 29: 917–932.

[38] Henton E, Meier-Augenstein W, Kemp HF (2010) The use of oxygen isotopes in sheep molars to investigate past herding practices at the Neolithic settlement of Catalhöyük, central Anatolia. Archaeometry 52: 429–449.

[39] Quilter J, Castillo Butters LJ (2010) New perspectives on Moche political organization. Washington D.C.: Dumbarton Oaks Research Library and Collections. 388p.

[40] Larco Hoyle R (1948) Cronología arqueológica del norte de Perú. Buenos Aires: Sociedad Geográfica Americana. 87 p.

[41] Peñaherrera Del Aguila C (1986) Gran geografía del Perú. Naturaleza y Hombre. Volumen I Geografía fisica del Perú. Lima: Manfer-Juan Mejia Baca. 220p.

[42] Collin Delavaud C (1968) Les régions côtières du Pérou septentrional. Lima: IFEA. 409p.

[43] Dollfus O (1978) Les Andes intertropicales : une mosaïque changeante. Annales ESC (Économies, Sociétés, Civilisations) 5–6: 895–905.

[44] Billman B (2002) Irrigation and the origins of the southern Moche State on the north coast of Peru. Latin American Antiquity 13: 371–400.

[45] Castillo Butters LJ (2010) Moche politics in the Jequetepeque valley. A case for Political Opportunism. In Quilter J, Castillo Butters LJ, editors. New perspectives on Moche political organization. Washington D.C.: Dumbarton Oaks Research Library and Collection. 83–109.

[46] Chauchat C, Gutiérrez León B (2006) Excavaciones en el Conjunto Arquitectónico 18 (Plataforma Uhle) durante las temporadas 1999 y 2000. In: Uceda S, Mujica E, Morales R, editors. Investigaciones en Huaca de la Luna 2000. Trujillo: Facultad de Ciencias Sociales-Universidad Nacional de la Libertad. 103–147.

[47] Chauchat C, Gutiérrez León B (2008) Excavaciones en la Plataforma Uhle, temporada 2001. In: Uceda S, Mujica E, Morales R, editors. Investigaciones en Huaca de la Luna 2001.Trujillo: Facultad de Ciencias Sociales-Universidad Nacional. 63–91.

[48] Chauchat C, Gutiérrez León B (2008) Excavaciones en la Plataforma Uhle, temporada 2002. In: Uceda S, Mujica E, Morales R, editors. Investigaciones en Huaca de la Luna 2002. Trujillo:Facultad de Ciencias Sociales-Universidad Nacional de la Libertad. 53–92.

[49] Chauchat C, Gutiérrez León B (2012) Excavaciones en la Plataforma Uhle durante el 2003: nuevas evidencias de entierros rituales Moche. In: Uceda S, Mujica E, Morales R, editors. Investigaciones en Huaca de la Luna 2003. Trujillo:Facultad de Ciencias Sociales-Universidad Nacional de la Libertad. 69–100.

[50] Chauchat C, Gutiérrez B, Deverly D, Goepfert N (2008) Recherches sur l’élite de la société mochica. La Plate-forme Uhle à Moche, sur la côte nord du Pérou. Les Nouvelles de l’Archéologie 111–112: 116–122.

[51] Chauchat C, Gutiérrez B, Deverly D, Goepfert N, Huchet JB (2009) La Plataforma Uhle en Moche: una síntesis de los descubrimientos. Revista del Museo de Arqueología, Antropología e Historia de Trujillo 11: 85–110.

[52] Franco R, Gálvez C, Vásquez S (1994) Arquitectura y decoración mochica en Huaca Cao Viejo, Complejo El Brujo: resultados preliminares. In: Uceda S, Mujica E, editors. Moche: propuestas y perspectivas. Actas del Primer Coloquio sobre la Cultura Moche (Trujillo, 12 al 16 de abril 1993). Lima: U.N.T.-IFEA y Asociación Peruana para el Fomento de las Ciencias Sociales. 147–180.

[53] Franco R, Gálvez C, Vásquez S (2005) El Brujo. Pasado Milenario. Trujillo: Ediciones Sian. 48 p.

[54] Franco R, Gálvez C, Vásquez S (2003) Modelos, función y cronología de la Huaca Cao Viejo, complejo El Brujo. In: Uceda S, Mujica E, editors. Moche. Hacia el final del milenio, Actas del Segundo Coloquio sobre la Cultura Moche (Trujillo, 1 al 7 de agosto de 1999). Trujillo: Pontificia Universidad Católica del Perú-Universidad Nacional de la Libertad. Vol. 2: 125–177.

[55] Franco R, Gálvez C, Vásquez S (1999) Tumbas de cámara Moche en la Plataforma Superior de la Huaca Cao Viejo, Complejo El Brujo. Lima: Fundación Augusto N. Wiese. 54p.

[56] Franco R, Gálvez C, Vásquez S (2001) Desentierro y Reenterramiento de una Tumba de Elite Mochica en el Complejo El Brujo. Lima: Fundación Augusto N. Wiese. 69p.

[57] Williams AR (2006) El misterio de la momia tatuada. National Geographic 19: 2–15.

[58] Kent JD (1982) The domestication and exploitation of the South American camelids: methods of analysis and their application to circum-lacustrine archaeological sites in Bolivia and Peru. PhD in Anthropology. Washington DC: Wasghinton University. 2 volumes.

[59] Wheeler JC (1982) Aging llamas and alpacas by their teeth. Llama World I: 12–17.

[60] O’Connell TC, Hedges RE, Healey MA, Simpson AHR (2001) Isotopic comparison of hair, nail and bone: Modern analyses. Journal of Archaeological Science 28: 1247–1255.

[61] Hedges REM, Clement JG, Thomas CDL, O’connell TC (2007) Collagen turnover in the adult femoral mid-shaft: Modeled from anthropogenic radiocarbon tracer measurements. American Journal of Physical Anthropology 133: 808–816. 17405135 10.1002/ajpa.20598

[62] Hillson (1986) Teeth. Cambridge: Cambridge University Press. 377 p.

[63] Balasse M (2002) Reconstructing dietary and environmental history from enamel isotopic analysis: Time resolution of intra-tooth sequential sampling. International Journal of Osteoarchaeology 12: 155–165.

[64] Passey BH, Cerling TE (2002) Tooth enamel mineralization in ungulates: Implications for recovering a primary isotopic time-series. Geochimica et Cosmochimica Acta 66: 3225–3234.

[65] Zazzo A, Balasse M, Passey BH, Moloney AP, Monahan FJ, et al (2010) The isotope record of short- and long-term dietary changes in sheep tooth enamel: Implications for quantitative reconstruction of paleodiets. Geochimica et Cosmochimica Acta 74: 3571–3586.

[66] Weinreb MM, Sharav DMD (1964) Tooth development in sheep. American Journal of Veterinary Research 25: 891–908. 14266896

[67] Wright LE, Schwarcz HP (1998) Stable carbon and oxygen isotopes in human tooth enamel: Identifying breastfeeding and weaning in prehistory. American Journal of Physical Anthropology 106: 1–18. 9590521 10.1002/(SICI)1096-8644(199805)106:1<1::AID-AJPA1>3.0.CO;2-W

[68] Longin R (1971) New method of collagen extraction for radiocarbon dating. Nature 230: 241–242. 4926713 10.1038/230241a0

[69] Bocherens H, Fizet M, Mariotti A, Lange-Badre B, Vandermeersch B, et al (1991) Isotopic biogeochemistry (^13^C, ^15^N) of fossil vertebrate collagen: application to the study of a past food web including Neandertal man. Journal of Human Evolution 20: 481–492.

[70] DeNiro MJ (1985) Postmortem preservation and alteration of in vivo bone collagen ratios in relation to palaeodietary reconstruction. Nature 317: 806–809.

[71] Van Klinken GJ (1999) Bone collagen quality indicators for palaeodietary and radiocarbon measurements. Journal of Archaeological Science 26: 687–695.

[72] Dobberstein RC, Collins MJ, Craig OE, Taylor G, Penkman KEH, et al (2009) Archaeological collagen: Why worry about collagen diagenesis? Archaeological and Anthropological Science 1: 31–42.

[73] Ambrose SH (1990) Preparation and characterization of bone and tooth collagen for isotopic analysis. Journal of Archaeological Science 17: 431–451.

[74] Koch PL, Tuross N, Fogel ML (1997) The effects of sample treatment and diagenesis on the isotopic integrity of carbonate in biogenic hydroxylapatite. Journal of Archaeological Science 24: 417–429.

[75] Shin JY, Hedges REM (2012) Diagenesis in bone and enamel apatite carbonate; the potential of density separation to assess the original composition. Journal of Archaeological Science 39: 1123–1130.

[76] Clementz MT, Fox-Dobbs K, Wheatley PV, Koch PL, Doak DF (2009) Revisiting old bones: coupled carbon isotope analysis of bioapatite and collagen as an ecological and palaeoecological tool. Geological Journal 44: 605–620.

[77] Zazzo A, Lécuyer C, Mariotti A (2004) Experimentally-controlled carbon and oxygen isotope exchange between bioapatites and water under inorganic and microbially-mediated conditions. Geochimica et Cosmochimica Acta 68: 1–12.

[78] Wang Y, Cerling TE (1994) A model of fossil tooth and bonediagenesis: Implications for paleodiet reconstruction from stable isotopes. Palaeogeography, Palaeoclimatology Palaeoecology 107: 281–289.

[79] DeNiro MJ, Epstein S (1978) Influence of diet on the distribution of carbon isotopes in animals. Geochimica et Cosmochimica Acta 42: 495–506.

[80] DeNiro MJ, Epstein S (1981) Influence of diet on the distribution of nitrogen isotopes in animals. Geochimica et Cosmochimica Acta 45: 341–351.

[81] Lee-Thorp JA, Sealy JC, Van Der Merwe NJ (1989) Stable carbon isotope ratio differences between bone collagen and bone apatite, and their relationship to diet. Journal of Archaeological Science 16: 585–599.

[82] Jim S, Ambrose SH, Evershed RP (2004) Stable carbon isotopic evidence for differences in the dietary origin of bone cholesterol, collagen and apatite: implications for their use in palaeodietary reconstruction. Geochimica et Cosmochimica Acta 68: 61–72.

[83] Fernandez R, Nadeau MJ, Grootes PM (2012) Macronutrient-based model for dietary carbon routing in bone collagen and bioapatite. Archaeological and Anthropological Sciences 4: 291–301.

[84] Ambrose SH, Norr L (1993) Experimental evidence for the relationship of the carbon isotope ratios of whole diet and dietary protein to those of bone collagen and carbonate. In: Lambert JB, Grupe G, editors. Prehistoric human bone Archaeology at the molecular level. Berlin: Springer. 1–37.

[85] Tieszen LL, Fagre T (1993) Effect of diet quality and composition on the isotopic composition of respiratory CO_2_, bone collagen, bioapatite, and soft tissues. In: Lambert JB, Grupe G, editors. Prehistoric human bone: archaeology at the molecular level. Berlin: Springer. 121–155.

[86] Warinner C, Tuross N (2009) Alkaline cooking and stable isotope tissue-diet spacing in swine: Archaeological implications. Journal of Archaeological Science 36: 1690–1697.

[87] Passey BH, Robinson TF, Ayliffe LK, Cerling TE, Sponheimer M, et al (2005) Carbon isotope fractionation between diet, breath CO_2_, and bioapatite in different mammals. Journal of Archaelogical Science 32 (10): 1459–1470.

[88] Cerling TE, Harris JM (1999) Carbon isotope fractionation between diet and bioapatite in ungulate mammals and implications for ecological and paleoecological studies. Oecologia 120: 347–363. 28308012 10.1007/s004420050868

[89] Smith BN, Epstein S (1971) Two categories of ^13^C/^12^C ratios for higher plants. Plant Physiology 47: 380–384. 16657626 10.1104/pp.47.3.380 PMC365873

[90] Ranson SL, Thomas M (1960) Crassulacean acid metabolism. Annual Review of Plant Physiology 11: 81–110.

[91] Tieszen LL, Chapman M (1992) Carbon and nitrogen isotopic status of the major marine and terrestrial resources in the Atacama Desert of northern Chile. In: Aufderheide A, editor. Proceedings of the First World Congress on Mummy Studies. Santa Cruz de Tenerife: Museo Arquelógico y Etnográfico de Tenerife. 409–425.

[92] Skrzypek G, Engel Z, Chuman T, Šefrna L (2011) *Distichia peat* - A new stable isotope paleoclimate proxy for the Andes. Earth and Planetary Science Letters 307: 298–308.

[93] Schoeninger MJ, DeNiro MJ (1984) Nitrogen and carbon isotopic composition of bone collagen from marine and terrestrial animals. Geochimica et Cosmochimica Acta 48: 625–639.

[94] Szpak P, Millaire JF, White CD, Longstaffe FJ (2012) Influence of seabird guano and camelid dung fertilization on the nitrogen isotopic composition of field-grown maize (*Zea mays*). Journal of Archaeological Science 39: 3721–3740.

[95] Ambrose SH, DeNiro MJ (1987) Bone nitrogen isotope composition and climate. Nature 325: 201. 10.1038/325201a0 3808017

[96] Ambrose SH (1991) Effects of diet, climate and physiology on nitrogen isotope abundances in terrestrial foodwebs. Journal of Archaeological Science 18: 293–317.

[97] Evans RD, Ehleringer JR (1994) Plant δ^15^N values along a fog gradient in the Atacama desert, Chile. Journal of Arid Environments 28: 189–193.

[98] Bocherens H, Drucker D (2003) Trophic level isotopic enrichment of carbon and nitrogen in bone collagen: case studies from recent and ancient terrestrial ecosystems. International Journal of Osteoarchaeology 13: 46–53.

[99] Minagawa M, Wada E (1984) Stepwise enrichment of ^15^N along food chains: further evidence and the relation between ^15^N and animal age. Geochimica et Cosmochimica Acta 48: 1135–1140.

[100] Schoeninger MJ, DeNiro MJ, Tauber H (1983) Stable nitrogen isotope ratios of bone collagen reflect marine and terrestrial components of prehistoric human diet. Science 220: 1381–1383. 6344217 10.1126/science.6344217

[101] Fuller BT, Fuller JL, Sage NE, Harris DA, O’Connell TC, et al (2004) Nitrogen balance and δ^15^N: why you’re not what you eat during pregnancy. Rapid Communication in Mass Spectrometry 18: 2889–2896. 10.1002/rcm.1708 15517531

[102] Fuller BT, Molleson TI, Harris DA, Hedges REM (2006) Isotopic evidence for breastfeeding and possible adult dietary differences from late/sub-Roman Britain. American Journal of Physical Anthropology129: 45–54. 10.1002/ajpa.20244 16229026

[103] Trueman CN, McGill RAR, Guyard PH (2005) The effect of growth rate on tissue-diet isotopic spacing in rapidly growing animals. An experimental study with Atlantic salmon (*Salmo salar*). Rapid Communication in Mass Spectrometry 19: 3239–3247. 10.1002/rcm.2199 16220502

[104] Katzenberg MA, Lovell NC (1999) Stable isotope variation in pathological bone. International Journal of Osteoarchaeology 9: 316–324.

[105] Mekota AM, Grupe G, Ufer S, Cuntz U (2006) Serial analysis of stable nitrogen and carbon isotopes in hair: monitoring starvation and recovery phases of patients suffering from anorexia nervosa. Rapid Communication in Mass Spectrometry 20: 1604–1610. 10.1002/rcm.2477 16628564

[106] Yacobaccio HD, Morales MR, Samec CT (2009) Towards an isotopic ecology of herbivory in the Puna ecosystem: new results and patterns on *Lama glama* . International Journal of Osteoarchaeology 19: 144–155.

[107] Schoeninger MJ, DeNiro MJ (1982) Carbon isotope ratios of apatite from fossil bone cannot be used to reconstruct diets of animals. Nature 297: 577–578. 7088140 10.1038/297577a0

[108] Marino BD, McElroy MB (1991) Glacial-to-interglacial variations in the carbon isotopic composition of atmospheric CO_2_ . Nature 349: 127–131.

[109] DeNiro MJ (1988) Marine food source for prehistoric coastal Peruvian camelids: isotopic evidences and implications. In: Wing ES, Wheeler JC, editors. Economic prehistory of the Central Andes. Oxford: British Archaeological Reports International Series 427 (BAR). 119–130.

[110] Longinelli A (1984) Oxygen isotopes in mammal bone phosphate: a new tool for paleohydrological and paleoclimatological research? Geochimica et Cosmochimica Acta 48: 385–390.

[111] Luz B, Kolodny Y, Horowitz M (1984) Fractionation of oxygen isotopes between mammalian bone-phosphate and environmental drinking water. Geochimica et Cosmochimica Acta 48: 1689–1693.

[112] Luz B, Kolodny Y (1985) Oxygen isotope variations in phosphate of biogenic apatites. IV. Mammal teeth and bones. Earth and Planetary Science Letters 75: 29–36.

[113] Amiot R, Lecuyer C, Buffetaut E, Fluteau F, Legendre S, et al (2004) Latitudinal temperature gradient during the Cretaceous Upper Campanian-Middle Maastrichtian: δ^18^O record of continental vertebrates. Earth and Planetary Science Letters 226: 255–272.

[114] Lécuyer C, Balter V, Martineau F, Fourel F, Bernard A, et al (2010) Oxygen isotope fractionation between apatite -bound carbonate and water determined from controlled experiments with synthetic apatites precipitated at 10°C to 37°C. Geochimica et Cosmochimica Acta 74: 2072–2081.

[115] White CD, Spence MW, Longstaffe FJ, Law KR (2000) Testing the nature of Teotihuacan imperialism at Kaminaljuyu Using Phosphate Oxygen-Isotope Ratios. Journal of Anthropological Research 56: 535–558.

[116] White CD, Spence MW, Longstaffe FJ, Stuart-Williams H, Law KR (2002) Geographic identities of the sacrificial victims from the Feathered Serpent Pyramid, Teotihuacan: Implications for the nature of state power. Latin American Antiquity 13: 217–236.

[117] Epstein S, Mayeda T (1953) Variation of ^18^O content of waters from natural sources Geochimica et Cosmochimica Acta. 4: 213–224.

[118] Dansgaard W (1964) Stable isotopes in precipitation. Tellus 16: 436–467.

[119] Bowen GJ, Wilkinson B (2002) Spatial distribution of δ^18^O in meteoric precipitation. Geology 30: 315–318.

[120] Gat JR (1996) Oxygen and hydrogen isotopes in the hydrologic cycle. Annual Review of Earth and Planetary Science 24: 225–262.

[121] White CD, Spence MW, Stuart-Williams K, Schwarcz HP (1998) Oxygen Isotopes and the Identification of Geographical Origins: The Valley of Oaxaca versus the Valley of Mexico. Journal of Archaeological Science 25: 643–655.

[122] Bowen GJ, Revenaugh J (2003) Interpolating the isotopic composition of modern meteoric precipitation. Water Resources Research 39: 1299.

[123] Sharp ZD (2007) Principles of stable isotope geochemistry. New Jersey: Pearson Prentice Hall: Upper Saddle River. 344p.

[124] IAEA/WMO (2006) Global network of isotopes in precipitation. The GNIP database. Available: http:/isohis.iaea.org.

[125] Roberts SB, Coward WA, Ewing G, Savage J, Cole TJ, et al (1988) Effect of weaning on accuracy of doubly labeled water method in infants. American Journal of Physiology-Regulatory, Integrative and Comparative Physiology 254: 622–627. 10.1152/ajpregu.1988.254.4.R622 3354710

[126] Wright LE, Schwarcz HP (1999) Correspondence between stable carbon, oxygen and nitrogen isotopes in human tooth enamel and dentine: infant diets at Kaminaljuyú. Journal of Archaeological Science 26: 1159–1170.

[127] Bonavia D (2008) El Maíz. Su origen, su domesticación y el rol cumplido en el desarrollo de la cultura. Lima: Universidad San Martin de Porres. 408p.

[128] Finucane BC, Maita Agurto P, Isbell WH (2006) Human and animal diet at Conchopata, Peru: stable isotope evidence for maize agriculture and animal management practices during the Middle Horizon. Journal of Archaeological Science 33: 1766–1776.

[129] Izeta AD, Laguens AG, Marconetto MB, Scattolin MC (2009) Camelid handling in the meridional Andes during the first millennium AD: a preliminary approach using stable isotopes. International Journal of Osteoarchaeology 19: 204–214.

[130] Balasse M, Tresset A, Dobney K, Ambrose SH (2005) The use of isotope ratios to test for seaweed eating in sheep. Journal of Zoology 266: 283–291.

[131] Yesner DR, Torres MJF, Guichon RA, Borrero LA (2003) Stable isotope analysis of human bone and ethnohistoric subsistence patterns in Tierra del Fuego. Journal of Anthropological Archaeology 22: 279–291.

[132] Verano JW, DeNiro MJ (1993) Locals or foreigners? Morphological, biometric and isotopic approaches to the question of group affinity in human skeletal remains recovered from unusual archaeological context. In: Sandford MK, editor. Investigations of Ancient Human Tissue: Chemical Analysis in Anthropology. Langhorne: Gordon and Breach. 361–386.

[133] Haas J, Creamer W, Huamán Mesíac L, Goldstein D, Reinharde K, et al (2012) Evidence for maize (*Zea mays*) in the Late Archaic (3000–1800 B.C.) in the Norte Chico region of Peru. Proceedings of the National Academy of Sciences of the United States of America 110: 4945–4949. 10.1073/pnas.1219425110 PMC3612639 23440194

[134] Johannessen S, Hastorf CA (1994) Corn and culture in the Prehistoric New World. Boulder: Westview press. 623p.

[135] Lambert PM, Gagnon CM, Billman BR, Katzenberg MA, Carcelén J, et al (2012) Bone Chemistry at Cerro Oreja: A stable isotope perspective on the development of a regional economy in the Moche Valley, Peru during the Early Intermediate Period. Latin American Antiquity 2012 23: 144.

[136] Gumerman G (1991) Subsistence and complex societies: diet between diverse socio-economic Groups at Pacatnamú, Peru. PhD in Anthropology. Los Angeles: University of California. 356 p.

[137] Gumerman G (1994) Corn for the dead: the significance of *Zea mays* in Moche burial offerings. In: Johannessen S, Hastorf CA, editors. Corn and Culture in the Prehistoric New World. Boulder: Westview press. 399–410.

[138] Gumerman G (1997). Botanical offerings in Moche burials at Pacatnamu. In: Donnan CB, Cock G, editors. The Pacatnamu Papers. Volume 2. Los Angeles: Fowler Museum of Cultural History-University of California. 243–249.

[139] Burger RL, Van der Merwe N (1990) Maize and the origin of Chavin civilization: an isotopic perspective. American Anthropologist 92: 85–95.

[140] McCorkle CM (1987) Punas, pastures and fields: grazing strategies and the agropastoral dialectic in an indigenous Andean community. In Browman DL, editor. Arid land use strategies and risk management in the Andes: A Regional Anthropological Perspective. Boulder: Westview Press. 57–80.

[141] Mengoni Goñalones GL (2007) Camelid management during Inca times in N.W. Argentina: models and archaeozoological indicators. Anthropozoologica 42: 129–141.

